# Molecular and cellular evolution of the amygdala across species analyzed by single-nucleus transcriptome profiling

**DOI:** 10.1038/s41421-022-00506-y

**Published:** 2023-02-14

**Authors:** Bin Yu, Qianqian Zhang, Lin Lin, Xin Zhou, Wenji Ma, Shaonan Wen, Chunyue Li, Wei Wang, Qian Wu, Xiaoqun Wang, Xiao-Ming Li

**Affiliations:** 1 Department of Neurobiology and Department of Neurology of the Second Affiliated Hospital, Zhejiang University School of Medicine, Hangzhou, Zhejiang China; 2 NHC and CAMS Key Laboratory of Medical Neurobiology, Ministry of Education Frontier Science Center for Brain Research and Brain-Machine Integration, School of Brain Science and Brain Medicine, Zhejiang University School of Medicine, Hangzhou, Zhejiang China; 3 State Key Laboratory of Brain and Cognitive Science, CAS Center for Excellence in Brain Science and Intelligence Technology, Institute of Biophysics, Chinese Academy of Sciences, Beijing, China; 4 University of Chinese Academy of Sciences, Beijing, China; 5 State Key Laboratory of Cognitive Neuroscience and Learning, Beijing Normal University, Beijing, China; 6 IDG/McGovern Institute for Brain Research, Beijing Normal University, Beijing, China; 7 Yunnan Key Laboratory of Primate Biomedical Research, Institute of Primate Translational Medicine, Kunming University of Science and Technology, Kunming, Yunnan China; 8 Research Units for Emotion and Emotion disorders, Chinese Academy of Medical Sciences, Beijing, China

**Keywords:** Transcriptomics, Bioinformatics

## Abstract

The amygdala, or an amygdala-like structure, is found in the brains of all vertebrates and plays a critical role in survival and reproduction. However, the cellular architecture of the amygdala and how it has evolved remain elusive. Here, we generated single-nucleus RNA-sequencing data for more than 200,000 cells in the amygdala of humans, macaques, mice, and chickens. Abundant neuronal cell types from different amygdala subnuclei were identified in all datasets. Cross-species analysis revealed that inhibitory neurons and inhibitory neuron-enriched subnuclei of the amygdala were well-conserved in cellular composition and marker gene expression, whereas excitatory neuron-enriched subnuclei were relatively divergent. Furthermore, *LAMP5*^+^ interneurons were much more abundant in primates, while *DRD2*^+^ inhibitory neurons and *LAMP5*^+^*SATB2*^+^ excitatory neurons were dominant in the human central amygdalar nucleus (CEA) and basolateral amygdalar complex (BLA), respectively. We also identified CEA-like neurons and their species-specific distribution patterns in chickens. This study highlights the extreme cell-type diversity in the amygdala and reveals the conservation and divergence of cell types and gene expression patterns across species that may contribute to species-specific adaptations.

## Introduction

The amygdala is responsible for animal survival due to its involvement in reproduction and fear/escape responses^1–4^. It also plays a critical role in emotional valence, memory encoding, feeding, social behaviors, and behavioral state assessment^5,6^. Damages or functional disruptions of the amygdala have been implicated in various neurological diseases, especially neuropsychiatric disorders, such as schizophrenia, anxiety, and bipolar disorder^7–9^. The amygdala is a heterogeneous complex that includes multiple subnuclei and neuronal cell types originating from cortical and subcortical territories^10^. In a landmark study^11^, Johnston suggested that the amygdala was composed of cortical-like and striatal-like components. Studies over the last few years have demonstrated that the amygdala is far more complex than previously thought. In addition to cortical and striatal (lateral ganglionic eminence; LGE) derivatives, the amygdala also contains neuronal cell types derived from the medial ganglionic eminence (MGE), caudal ganglionic eminence (CGE), preoptic area (POA), and even supraoptoparaventricular hypothalamic domain (SPV)^12–14^. Given its multiple embryonic origins, the amygdala is a mosaic-like structure with an abundance of neuronal cell types. However, our understanding of the cellular composition and gene expression patterns of the amygdala remains limited, though two hundred years have passed since^15^ first described this almond-shaped mass of gray matter. Notably, the lack of cell-type-specific studies has been a major obstacle to dissecting amygdala function and understanding amygdala-related disorders.

Although the amygdala is highly complex in terms of neuronal subtypes, molecular profiles, and connections, growing evidence suggests that primary amygdala subnuclei and basic circuit connections and functions are conserved across species, especially in mammals and sauropsids^14,16–18^. They share a common pattern of organization and the same basic combinations of developmental regulatory genes that orchestrate similar functions, although species differences do exist. The relative size and complexity of the amygdala subnuclei differ among species^19–21^. The paralaminar nucleus (PL) of the amygdala is relatively enlarged in humans and non-human primates compared to other mammals^22^. Localization of amygdala subnuclei in sauropsids remains controversial^17^. However, the extent to which evolution has changed cell types and gene expression patterns of the amygdala across species is still unclear.

Recent advances in single-cell and single-nucleus RNA-sequencing (scRNA-seq/snRNA-seq) technologies have facilitated the molecular characterization of diverse cell types in different brain regions across species^23–27^. Although single-cell profiling of the amygdala in different species has been reported, the number of neurons previously captured in the human amygdala was too small to allow effective clustering and in-depth analysis of neurons^28^, amygdala subnuclei, which varied greatly in cell types and physiological functions were not distinguished in scRNA-seq/snRNA-seq in primates^28–30^, and inconsistent dissection of amygdala regions also hindered cross-species comparison^30,31^. To ensure the consistency of dissection regions, obtain high quality and unbiased sequencing data, and explore the evolutionary conservation and divergence of the amygdala, we applied single-nucleus transcriptomic methods for profiling human, macaque, mouse, and chicken amygdala homologs.

## Results

### Taxonomy of cell types in human, macaque, and mouse amygdala

**Fig. 1:**
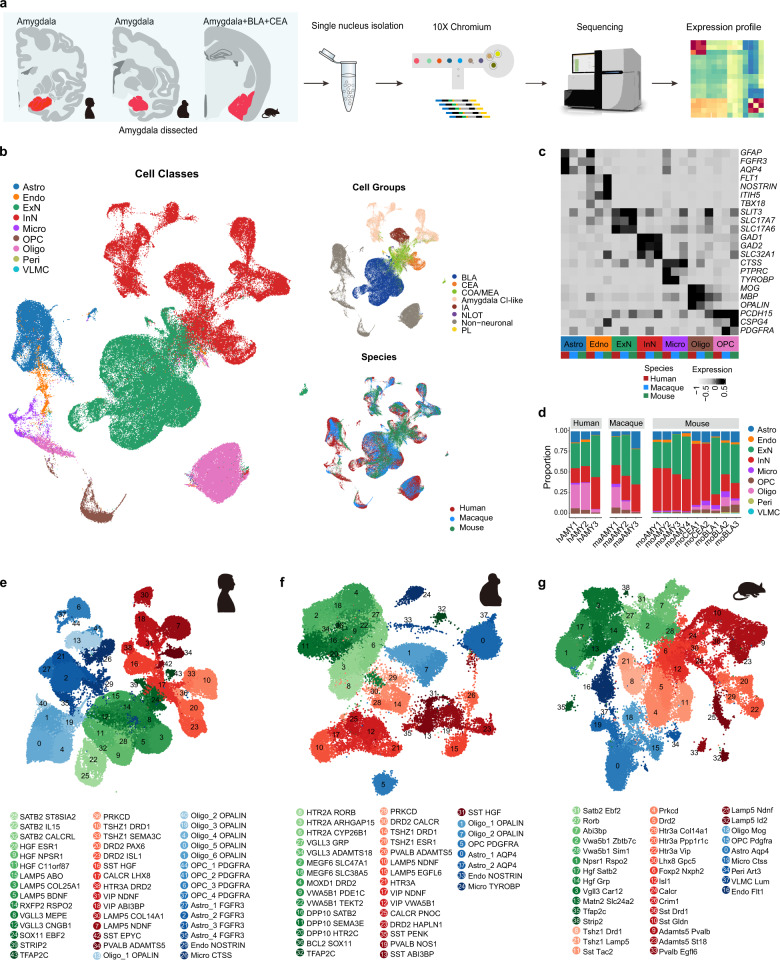
Profiling of amygdala cell types across mammals by snRNA-seq. **a** Schematic showing overall study design. 10× Genomics experimental workflow was applied to nuclei isolated from human, macaque, and mouse amygdala. **b** Uniform manifold approximation and projection (UMAP) plot of integrated human, macaque, and mouse datasets, with nuclei colored by cell class, amygdala cell group, and species. The total number of nuclei was 171,152. Astro astrocytes, Endo endothelial cells, ExN excitatory neurons, InN inhibitory neurons, Micro microglia, Oligo oligodendrocytes, OPC oligodendrocyte precursor cells, Peri pericytes, VLMC vascular leptomeningeal cells, Amygdala CI-like amygdala cortical interneuron-like inhibitory cells, BLA basolateral amygdalar complex, CEA central amygdalar nucleus, COA/MEA cortical amygdalar area/medial amygdalar nucleus, IA intercalated amygdalar nucleus, NLOT nucleus of the lateral olfactory tract, Non-neuronal non-neuronal cells, PL paralaminar nucleus. **c** Heat map showing average expression of canonical marker genes in cell classes, separated by species. **d** Percentage of cell classes across humans, macaques, and mice. Cell classes are colored as in **b**. hAMY human amygdala; maAMY macaque amygdala; moAMY mouse amygdala; moBLA mouse BLA; moCEA mouse CEA. **e**–**g** UMAP plots of 39 clusters from mouse datasets (**e**), 38 clusters from macaque datasets (**f**), and 45 clusters from human datasets (**g**). See also Supplementary Figs. S1 and S2.

To characterize cell types of the amygdala in mammals and sauropsids (the most diverse and successful terrestrial vertebrates) at the transcriptomic level, we applied snRNA-seq assays (Chromium v3) using human, macaque, mouse, and chicken samples (Fig. 1a). Human amygdala samples were obtained from postmortem donors within a very short postmortem interval (within 6 h), and macaque, mouse, and chicken amygdala samples were obtained from freshly dissected specimens. We profiled a total of 17 specimens, including three human amygdala samples, three macaque amygdala samples, four mouse amygdala samples, three mouse BLA (containing lateral (LA), basal (BA), basomedial (BM), and posterior (PA) nuclei) samples, two mouse CEA samples, and two chicken amygdala homolog samples (Supplementary Table S1). After quality control and filtering, we obtained 203,971 high-quality single nuclei from the four species. 91,699 nuclei with a median of 9417 unique molecular identifiers (UMIs) and 3617 genes were from the human dataset, 45,626 nuclei with a median of 4631 UMIs and 2373 genes were from the macaque dataset, 54,186 nuclei with a median of 5020 UMIs and 2316 genes were from the mouse dataset, and 12,460 nuclei with a median of 3660 UMIs and 1716 genes were from the chicken dataset (Supplementary Fig. S1a, b and Table S1).

To evaluate conservation and divergence across species, we first integrated amygdala RNA-seq data from mammals using Seurat (v4.0.5). Integration revealed broad conservation of cell types across mammals, as nuclei from different species were well mixed (Fig. 1b). Seven cell classes were recognized based on the expression of known cell class markers: i.e., excitatory neurons (ExN), inhibitory neurons (InN), oligodendrocyte precursor cells (OPC), oligodendrocytes (Oligo), astrocytes (Astro), endothelial cells/pericytes (Endo/Peri), and microglia (Micro) (Fig. 1b, c; Supplementary Fig. S1c). Analysis of differentially expressed genes (DEGs) in each cell class revealed shared DEGs across species, although many showed enriched expression in only one species (Supplementary Fig. S1d). Interestingly, ExN shared a lower percentage of DEGs across species than InN, and non-neuronal cells shared a greater percentage of DEGs across species than neuronal cells (Supplementary Fig. S1d), implying that neurons, especially ExN, were evolutionarily more diverse. We also identified many other ExN and InN markers in the mammalian amygdala in addition to *SLC17A7*, *GAD1*, and *GAD2*, such as *C1QL3* for ExN, and *ERBB4* and *IGF1* for InN (Supplementary Fig. S1e). Remarkably, more marker genes were identified in the InN that were conserved among species than in the ExN, again suggesting higher ExN diversity in the amygdala (Supplementary Fig. S1e).

An unprecedented number of mammalian amygdala neuronal and non-neuronal cells were captured in our datasets. Strikingly, the vast majority were neuronal cells (52,650 nuclei in the human dataset, 32,525 nuclei in the macaque dataset, and 43,654 nuclei in the mouse dataset), with ExN and InN accounting for about 50% each (Fig. 1d; Supplementary Fig. S1f). In contrast, InN constitute ~15%–30% of neuronal cells in the neocortex of different species^26^, consistent with the diversity of InN in the amygdala. Notably, primates possessed slightly more ExN, while mice possessed more InN, in agreement with the expanded ExN-enriched BLA structure in primates^21^. In addition, consistent with white matter expansion during primate evolution^32^, we found that primates, especially humans, contained more oligodendrocyte precursor cells and oligodendrocytes (Supplementary Fig. S1f).

Unsupervised clustering identified 45, 38, and 39 distinct transcriptomic cell types in the human, macaque, and mouse amygdala, respectively (Fig. 1e–g; Supplementary Fig. S2 and Table S2). We assigned each cluster a name based on the marker gene showing the most specific expression in that cluster (Fig. 1e–g; Supplementary Fig. S2). In most clusters, two genes were used to define a cell type. Some cluster names were manually adjusted based on knowledge for consistency of nomenclature across species. We found that some widely reported cell types in the mouse amygdala were captured in our mouse dataset. For example, the *Prkcd*, *Sst Tac2*, and *Drd2* clusters, which likely originated from the CEA^33–35^, and the *Tshz1* clusters, which likely originated from the intercalated amygdalar nucleus (IA)^36^, occupied the same branches on the dendrogram (Supplementary Fig. S2). The *Npsr1 Rspo2* and *Hgf Grp* clusters probably correspond to the *Rspo2*^*+*^ and *Grp*^*+*^ neurons reported previously^37,38^. Notably, assumed homologous cell types, e.g., the *PRKCD*, *DRD2*, and *TSHZ1* clusters, were also identified in the human and macaque datasets and were clustered together on the dendrogram (Supplementary Fig. S2), indicating general conservation across species.

### Evolutionary conservation and divergence of amygdala subnuclei in mammals

**Fig. 2:**
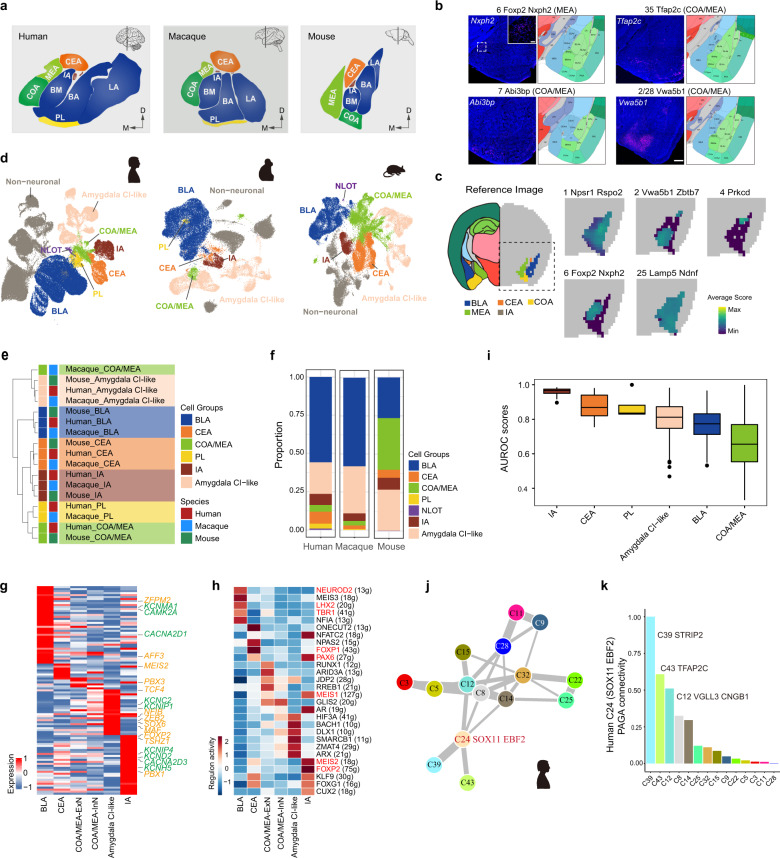
Evolutionary conservation and divergence of amygdala subnuclei in mammals. **a** Anatomical localization of amygdala subnuclei in each species. Coronal sections of Allen Brain Reference Atlases (https://portal.brain-map.org/) and Atlas of the rhesus monkey brain^101^ were used as references. BA basal nucleus, BM basomedial nucleus, CEA central amygdalar nucleus, COA cortical amygdalar area, IA intercalated amygdalar nucleus, LA lateral nucleus, MEA medial amygdalar nucleus, PL paralaminar nucleus. **b** Spatial expression patterns of mouse cell-type marker genes in mouse amygdala, confirmed by mFISH analysis. Example coronal sections are shown, scale bar: 300 μm. Insets, magnifications of indicated region, scale bar: 50 μm. **c** Spatial expression patterns of top five differentially expressed genes (DEGs) of selected mouse cluster in amygdala. Left panel image is the reference coronal section at slice 37 of Allen Brain Reference Atlas. For every selected cluster, average score of top five DEGs in each space voxel was depicted using colormap. **d** UMAP embedding of datasets from human, macaque, and mouse amygdala, with nuclei colored by cell group based on expression patterns of marker genes, Ro/e analysis, and prediction algorithm. Amygdala CI-like amygdala cortical interneuron-like inhibitory cells; BLA basolateral amygdalar complex; CEA central amygdalar nucleus; COA/MEA cortical amygdalar area/medial amygdalar nucleus; IA intercalated amygdalar nucleus; NLOT nucleus of the lateral olfactory tract; Non-neuronal non-neuronal cells; PL paralaminar nucleus. **e** Unsupervised hierarchical clustering of amygdala cell groups from different species based on area under the receiver operator characteristic curve (AUROC) scores reported by MetaNeighbor. **f** Bar plot showing proportion of nuclei from each cell group in each species. **g** Heat map showing conserved signature genes for each amygdala cell group. Genes labeled on right in orange encode transcription factors (TFs); genes in green encode ion channels. COA/MEA-ExN excitatory neurons from COA/MEA group; COA/MEA-InN inhibitory neurons from COA/MEA group. **h** Heat map of TF regulon activity for each amygdala cell group. Regulon gene set names are labeled on right and number of genes in each gene set is written in round brackets. Major regulon genes shown in red: genes that were already known to be highly expressed or important for subnuclei specialization during amygdala development. **i** Box plot showing total AUROC scores from each expression-based cluster grouped by cell-group origin (shown in Supplementary Fig. S4b). Center line, box bounds, and whiskers represent mean, 25th to 75th percentile range, and minimum to maximum range, respectively. Cell groups are colored as in Fig. 2e. **j** PAGA graph showing inferred developmental trajectories of human excitatory neuronal clusters. Line width represents strength of connectivity between two clusters. **k** Bar plot showing PAGA connectivity with Cluster 24 (C24 SOX11 EBF2) in human dataset. See also Supplementary Figs. S3 and S4.

The amygdala consists of multiple subnuclei with various neuronal cell types originating from different cortical and subcortical territories. Anatomically, the amygdala subnuclei are well defined in mammals (Fig. 2a). To determine subnuclear localization of each neuronal cell type, we first applied in situ hybridization (ISH) data from the Allen Brian Atlas (ABA)^39^ and performed RNAscope multiplex fluorescent ISH (mFISH) to validate the expression patterns of the most specific marker genes of each cluster in the mouse amygdala (Fig. 2b; Supplementary Fig. S3a). Furthermore, using coordinated information and expression levels of genes in the ABA, we further analyzed the mean expression level and patterns of the top five marker genes in each cluster in the mouse amygdala (Fig. 2c; Supplementary Fig. S3b). Finally, we preformed enrichment analysis to identify BLA- or CEA-enriched clusters based on the anatomical origin of each neuronal nucleus (Supplementary Fig. S3c). Analysis clearly showed that neurons in each cluster exhibited spatial distribution specificity and were mainly confined in one subnucleus of the amygdala. Based on the expression patterns of marker genes, cluster dendrograms, and knowledge, we divided the mouse neuronal cell types into six groups: BLA-derived excitatory neurons (BLA group), CEA-derived inhibitory neurons (CEA group), IA-derived inhibitory neurons (IA group), cortical amygdalar area/medial amygdalar nucleus-derived excitatory and inhibitory neurons (COA/MEA group), amygdala cortical interneuron-like inhibitory neurons (Amygdala CI-like group), and nucleus of the lateral olfactory tract-derived excitatory neurons (NLOT group). To assign cell group information to human and macaque neuronal cell types, we used Seurat label transfer to predict the group origin of every neuronal cell based on transcriptomic similarity with the mouse dataset (Supplementary Fig. S3d, e). Except for the NLOT group neurons in the macaque dataset, we captured all subnucleus-derived cell types in the mammalian amygdala after prediction and manual adjustment, including primate-specific PL-derived cell types (PL group), which show high expression of immature marker genes^29^. As NLOT group neurons were not captured in our macaque dataset and given the continued debate over whether the NLOT is part of the amygdala, the NLOT was not included in further analyses. Remarkably, nuclei from the same cell group were clustered together in the uniform manifold approximation and projection (UMAP) plots, indicating similarity in gene expression between neurons from the same cell group (Fig. 2d; Supplementary Fig. S3d, e).

Hierarchical clustering of cell groups and cell types revealed general conservation of the amygdala across mammals, although the COA/MEA group neurons showed relatively high divergence (Fig. 2e; Supplementary Fig. S4b). The CEA group, IA group, and Amygdala CI-like group neurons showed similar proportions and diversity in the amygdala across species. However, the proportion of BLA group neurons was approximately two-fold higher in the primate amygdala than in the mouse amygdala, and more cell types were captured in primates. Conversely, mice exhibited a higher proportion and diversity of COA/MEA group neurons (Fig. 2f; Supplementary Fig. S4a). These results are consistent with the different relative sizes and cell densities of the BLA and COA/MEA across species, which may reflect species-specific adaptations^21^. We next identified cell group-level markers. Several marker genes were conserved across species, including some transcription factors and ion channel-encoding genes, but most markers were enriched in only one species (Fig. 2g; Supplementary Fig. S4c and Table S3). Consistent with the hierarchical clustering results, the COA/MEA group had fewer conserved marker genes (Fig. 2g). Furthermore, specifically activated regulons in each cell group were identified, which may be important for subnuclear specialization during development (Fig. 2h). For example, NEUROD2 is a neuronal basic helix-loop-helix transcription factor that is necessary for neurogenic differentiation and synaptic maturation^40,41^. Previous studies have shown that *Neurod2* is essential for LA and BA formation during development, as well as normal amygdala function such as emotional learning^42^. Our results indicated that NEUROD2 regulon activity was enriched in the BLA excitatory neurons, further underscoring the importance of *NEUROD2* and related regulatory genes for BLA formation and function. Many major regulon genes, such as *LHX2*, *TBR1*, *PAX6*, *FOXP1*, *MEIS1*, *MEIS2*, and *FOXP2*, have been implicated in subnuclear specialization and normal physiological function of the amygdala^14,43,44^, thus demonstrating the reliability of our regulon analysis. Importantly, we identified many novel major genes and regulons that may play critical roles in the specialization of specific cell groups (Fig. 2h).

To evaluate differences in evolutionary conservation of amygdala subnuclei, we performed unsupervised MetaNeighbor analysis. Results showed that the IA and CEA group neurons were the most evolutionarily conserved, while the BLA and COA/MEA group neurons were relatively evolutionarily divergent (Fig. 2i). Consistent with the MetaNeighbor results, analysis of the expression patterns of orthologous genes across species demonstrated that the BLA and COA/MEA groups had the greatest divergent expression (Supplementary Fig. S4d). In addition, fewer divergent genes were identified between primates than between humans and mice, suggesting that amygdala homology decreases with evolutionary distance (Supplementary Fig. S4e).

It should be noted that humans and macaques contained a neuronal cluster with high expression of immature markers, such as *SOX11* and *BCL2*, which were likely derived from the PL^22,29^. The PL is expanded in primates, containing numerous immature neurons even in adults, and is believed to play an important role in neuroplasticity in the adult brain^22^. PAGA developmental trajectory analysis indicated that the maturation trajectory of the PL-derived cluster in humans and macaques may be towards the VGLL3 and TFAP2C clusters (Fig. 2j, k; Supplementary Fig. S4f–i).

### Homology of inhibitory neurons in mammals

**Fig. 3:**
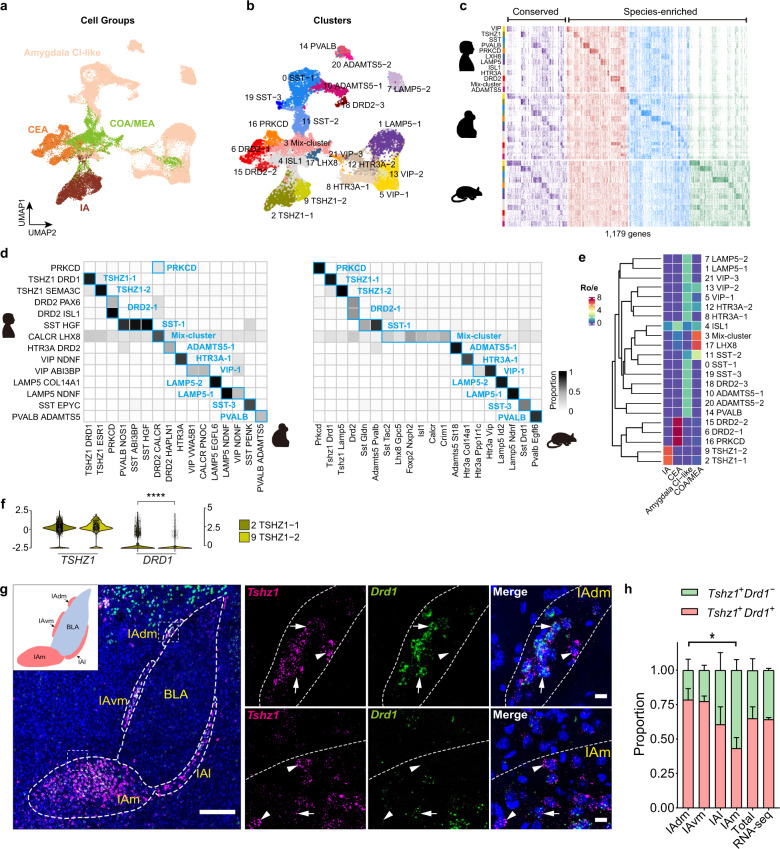
Homology of inhibitory neurons in mammals. **a**, **b** UMAP embedding of integrated inhibitory neurons from human, macaque, and mouse snRNA-seq datasets (using Seurat), with nuclei colored by **a** cell group and **b** annotated cell cluster. Amygdala CI-like amygdala cortical interneuron-like inhibitory cells; CEA central amygdalar nucleus; COA/MEA cortical amygdalar area/medial amygdalar nucleus; IA intercalated amygdalar nucleus. **c** Heat map showing expression of conserved (171 genes) and species-enriched (1008 genes) DEGs, ordered by major cell type and species. **d** Proportion of nuclei overlapping between human and macaque clusters or between human and mouse clusters in integrated datasets. Homologous clusters are labeled. **e** Dendrogram showing hierarchical clustering of inhibitory neuronal cell types based on average expression of highly variable genes (HVGs) (by *hclust* function). Heat map showing the cell group enrichment of each cell type cluster estimated by Ro/e score. Ro/e score referred to the ratio of observed to expected nuclei numbers in each cluster across cell groups. The higher the value of Ro/e, the greater enrichment in corresponding cell group. **f** Violin plots showing expression of selected genes in *TSHZ1-1* and *TSHZ1-2* clusters. Wilcoxon test was used for significance comparison (****P-value < 2.22e−16). **g** mFISH analysis of IA marker genes *Tshz1* and *Drd1* in mouse amygdala. Example coronal section is shown, scale bar: 200 μm. Boxed regions are enlarged at right and split into single-channel images. Arrows indicate *Tshz1*^+^ *Drd1*^+^ cells, arrowheads indicate *Tshz1*^+^ *Drd1*^−^ cells, scale bar: 10 μm. Note that co-labeled neurons tended to be distributed in the center of each IA subregion, while *Tshz1* single-positive neurons tended to be distributed in the periphery of each IA subregion. IAdm dorsomedial part of paracapsular island of IA; IAl lateral part of paracapsular island of IA; IAm main island of IA; IAvm ventromedial part of paracapsular island of IA. **h** Proportion of *Tshz1*^+^ *Drd1*^+^ and *Tshz1*^+^ *Drd1*^−^ neurons in different parts of IA and mouse snRNA-seq datasets. Analysis of variance (ANOVA) followed by Tukey’s HSD test were used for multiple comparisons (*P-value < 0.05). Data are represented as means ± SEM. See also Supplementary Figs. S5 and S6.

Abundant inhibitory neurons were enriched in the CEA, IA, and COA/MEA (mainly MEA), while CI-like inhibitory neurons were sparsely distributed in the amygdala. To further assess neuronal homology in the amygdala, we integrated the inhibitory neuron datasets of the mammals. Inhibitory neurons were well mixed across species and same cell group-derived nuclei were clustered together in the UMAP plots (Fig. 3a; Supplementary Fig. S5a). We identified 12 major cell types that were well aligned across species, except for one major cell type whose nuclei were predominately derived from the mouse MEA (named Mix-cluster) (Fig. 3b). Consistent with the motor cortex^26^, the expression of several marker genes in each major cell type was conserved across species, but most marker genes were enriched in only one species (Fig. 3c; Supplementary Table S4). We next identified 22 homologous cell types at appropriate resolution (Fig. 3b). Cluster membership shared among species, hierarchical clustering of homologous cell types, and MetaNeighbor analysis of cell types from humans, macaques, and mice further revealed cross-species conservation of inhibitory neurons, although differences across species did exist (Fig. 3d, e; Supplementary Fig. S5b, c). Inhibitory neuron homology between humans and macaques was higher than that between humans and mice, reflecting differences in evolutionary distance (Supplementary Fig. S5d).

CEA- and IA-derived inhibitory neurons are interesting because their LGE origin is distinct from other amygdala inhibitory neurons and cortical interneurons, which are mainly derived from the MGE and CGE, constituting the amygdala’s main output and inhibitory control center^14,45^. Previous studies have illustrated that the CEA and IA represent the most primitive and evolutionarily conserved entities in the amygdala, and likely existed in the last common ancestor of tetrapods^14,17^. The evolutionary conservation of these subnuclei is crucial for animal survival because they are involved in mediating many autonomic and endocrine responses in fear and anxiety behaviors^5,46^. Our snRNA-seq datasets confirmed the conservation of CEA and IA subnuclei at the cell-type level in mammals. Notably, the IA subnuclei were highly conserved, and all mammals in our datasets contained two types of *TSHZ1*^+^ neurons, which matched one-to-one between species (Fig. 3d). Studies have shown that IA-derived inhibitory neurons highly express *DRD1* and are densely innervated by dopaminergic afferents, providing feedforward inhibition of the BLA and CEA^47,48^. Interestingly, in our datasets, only one type of *TSHZ1* cluster highly expressed *DRD1*, which was consistent across species (Fig. 3f). Therefore, we speculated that there were two types of neurons in the IA, i.e., *Tshz1*^*+*^ *Drd1*^+^ and *Tshz1*^*+*^ *Drd1*^−^ neurons. This speculation was confirmed by mFISH analysis, which showed that both types of neurons were present in the main and paracapsular islands of the IA, and *Tshz1*^*+*^ *Drd1*^*−*^ neurons tended to be distributed in the IA periphery. Moreover, *Tshz1*^*+*^ *Drd1*^*−*^ neurons primarily resided in the main island of the IA, while *Tshz1*^*+*^ *Drd1*^+^ neurons dominated in the medial paracapsular island of the IA (Fig. 3g, h). Overall, mFISH results showed two IA cell types in mice, one expressing *Drd1* and the other not, which were probably well-conserved in mammals.

Despite broad conservation, differences among species were also observed. *LAMP5*^+^ neurons were classified into the CI-like group and likely shared the same derivation as cortical *LAMP5*^+^ interneurons. In accordance with recent observations that *LAMP5*^+^ interneurons are expanded throughout the neocortex in primates^25,45^, the proportion of *LAMP5*^+^ inhibitory neurons was approximately two to five times higher in the primate amygdala than in the mouse amygdala (Supplementary Fig. S5e–g). The expansion of *LAMP5*^+^ inhibitory neurons may be a common feature of the cerebral cortex and cerebral nuclei, and its evolutionary implications need to be further explored. *DRD2*^+^ neurons were much more abundant in humans than in macaques and mice and were segregated into two cell types (Supplementary Fig. S5e–g and Table S5) expressing *PAX6* and *ISL1*, respectively (Supplementary Fig. S5h). The former is a marker of the dorsal LGE, and the latter is a marker of the ventral LGE^49^. Gene Ontology (GO) analysis implied that the two types of *DRD2*^+^ neurons may regulate different physiological functions and behaviors through different signaling pathways (Supplementary Fig. S5i). Given the importance of *DRD2* and the dopamine system in human sociality and psychiatric disorders^50,51^ and specific increased abundance of *DRD2*^+^ neurons in the human amygdala, it is important to study the function of *DRD2*^+^ neurons in the human amygdala directly.

To identify both evolutionarily conserved and species-specific sets of co-regulated genes across cell types, we performed Coordinated Gene Activity in Pattern Sets (CoGAPS) and transfer-learning analyses (projectR). CoGAPS analysis allowed unsupervised identification of patterns of gene sets (Patterns) that represented common features across cell types, and projectR was used to project the snRNA-seq expression matrix into the CoGAPS pattern amplitude matrix. In general, broadly similar cellular expression patterns in similar cell types were identified across species (Supplementary Fig. S6a and Table S6). For example, “Pattern-66” was strongly correlated with IA (TSHZ1) clusters in all datasets (Supplementary Fig. S6b), and many IA regulators and marker genes were among the top 30 weighted genes in “Pattern-66”, including *FOXP2*, *NRG1*, *PBX3*, *MEIS2*, and *ERBB4* (Supplementary Fig. S6c)^44,52^. However, many Patterns showed high correlations among incongruent cell types across species, which may contribute to species-specific functions.

### Shared and distinct signatures of BLA excitatory neurons in mammals

**Fig. 4:**
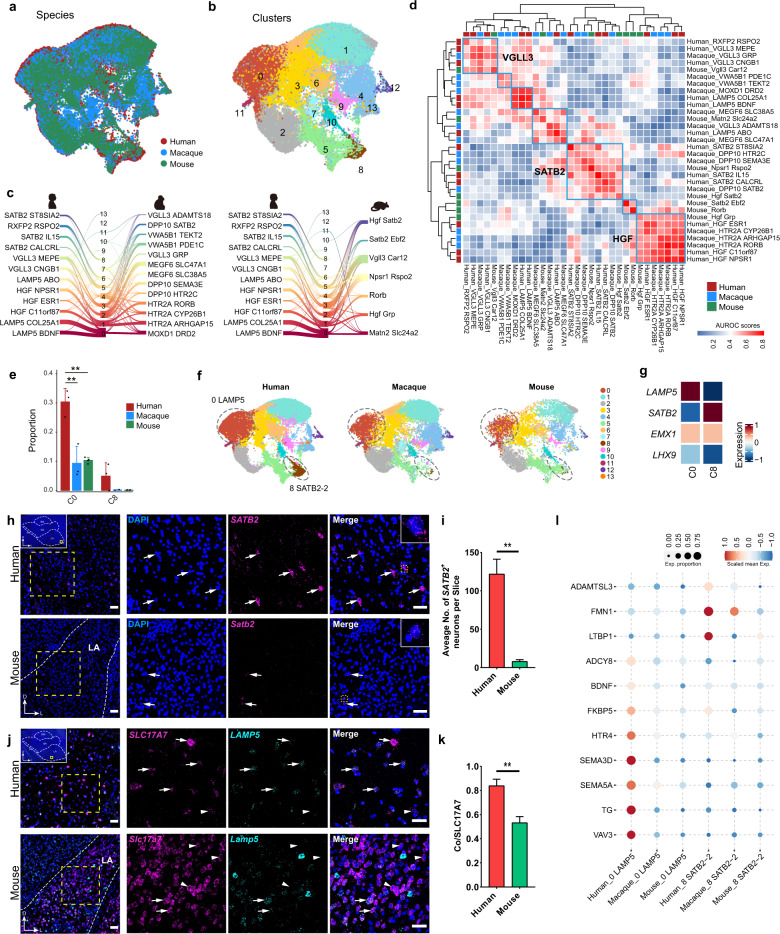
Shared and distinct signatures of BLA excitatory neurons in mammals. **a**, **b** UMAP embedding of integrated BLA excitatory neuronal datasets (using Seurat), with nuclei colored by species (**a**) and cluster (**b**). **c** River plots showing origin of integrated cell types. Thickness of line represents proportion of cells from human and macaque original clusters or from human and mouse original clusters within integrated cell types. Lines were cut off with a fraction < 0.035. **d** Heat map showing AUROC scores (estimated by MetaNeighbor) for BLA excitatory neuronal cell types from three mammals. AUROC scores were used to assess similarities across each pair of cell types. Cell types with high transcriptomic similarity across three species are marked by blue lines, *VGLL3*, *SATB2*, and *HGF* clusters were highly conserved across species. **e** Relative proportions of nuclei in selected cell types. ANOVA followed by Tukey’s HSD test were used for multiple comparisons (**adjusted *P*-value < 0.01). Data are represented as means ± SEM. **f** UMAP integrated BLA excitatory neuronal datasets, separated by species and cell type colored as in **b**. Cell types circled by dotted lines are human-dominant clusters. **g** Heat map showing expression of selected genes in C0 (*LAMP5*) and C8 (*SATB2-2*) clusters, which specifically expressed dorsal and lateral pallial marker gene *EMX1*, but not ventral pallial marker gene *LHX9*. **h** mFISH analysis of *SATB2* gene in human and mouse LA. Example coronal sections are shown, scale bar: 50 μm. Insets, coronal section of entire human amygdala and imaging location. Boxed regions are enlarged at right and split into single-channel images. Arrows indicate *SATB2*^+^ cells, scale bar: 30 μm. **i** Quantification of *SATB2*^+^ cells in human and mouse LA. Four human and mouse slices were used for quantification. Unpaired *t*-test was used for significance comparison (**P-value < 0.01). Data are represented as means ± SEM. **j** mFISH analysis of *SLC17A7* and *LAMP5* genes in human and mouse LA. Example coronal sections are shown, scale bar: 50 μm. Insets, coronal section of entire human amygdala and imaging location. Boxed regions are enlarged at right and split into single-channel images. Arrows indicate *SLC17A7*^+^ *LAMP5*^+^ cells, arrowheads indicate *SLC17A7*^+^ *LAMP5*^−^ cells, scale bar: 30 μm. **k** Proportion of *SLC17A7* and *LAMP5* double-positive cells in total *SLC17A7*-positive cells in human and mouse LA. Three human and four mouse slices were used for quantification. Unpaired *t*-test was used for significance comparison (**P-value < 0.01). Data are represented as means ± SEM. **l** Bubble plot showing that genes associated with psychiatric disorders were highly expressed in human-dominant cell types *LAMP5* and *SATB2-2*. See also Supplementary Figs. S7 and S8.

The BLA is the largest subnucleus in the amygdala, contains the most excitatory neurons, and is extensively connected to cortical and subcortical areas, especially primary sensory areas. Hence, the BLA is thought to receive information inputs and transmit decoded information to output subnuclei of the amygdala or other downstream brain areas^5^. Evolutionarily, the relative enlargement of the BLA compared with other subnuclei suggests it may have undergone the most significant evolutionary changes^19,21^. To assess BLA homology, we integrated the mammalian BLA-derived excitatory neurons and identified 14 cross-species cell types (Fig. 4a, b; Supplementary Fig. S7a). The BLA excitatory neurons showed higher interspecies heterogeneity than the inhibitory neurons, as reflected by the integrated data having more undefinable cell types and more species-dominant cell types (Fig. 4c; Supplementary Fig. S7b).

Despite variation among species, homologous major cell types were also identified in the BLA excitatory neurons. MetaNeighbor analysis and proportion of overlapping nuclei between clusters in the integrated datasets indicated that SATB2, HGF, and VGLL3 neuronal cell types were highly conserved in mammals (Fig. 4d; Supplementary Fig. S7c). Among them, VGLL3 cells were the most conserved BLA excitatory neurons, with at least one corresponding cell type found in each species (Fig. 4d; Supplementary Fig. S7c). CoGAPS analysis further identified highly correlated gene set Patterns in homologous cell types between species (Supplementary Fig. S7d, e and Table S7). Pattern-83 was strongly correlated with *VGLL3*^*+*^ cell types in all three mammalian datasets (Supplementary Fig. S7e). We also detected average expression of the top 30 weighted genes in Pattern-83 in human, macaque, and mouse BLA excitatory neuronal cell types, and found that gene expression was indeed enriched in the *VGLL3*^+^ neuronal cell types in each species (Supplementary Fig. S7f). Notably, *ESR1* was one of the most influential genes in Pattern-83 (Supplementary Fig. S7f). *Esr1* is highly expressed in the posterior amygdalar nucleus (PA) in the mouse brain, consistent with the expression pattern of the *Vgll3 Car12* cluster marker gene *Car12* (Supplementary Fig. S7g). *Esr1*^+^ neurons in the PA play key roles in both male aggression and reproduction by targeting the medial preoptic nucleus (MPN) and ventrolateral part of the ventromedial hypothalamus (VMHvl), respectively^53^. *Esr1* encodes estrogen receptor alpha, and estrogen signaling in other brain regions mediates instinctive social behaviors such as aggression, defense, and maternal behaviors^54–56^. Therefore, we speculate that *VGLL3*^+^ neurons are important for instinctive behaviors and conservation of these high *ESR1*-expressing clusters across species may have been critical for animal survival and reproduction during evolution.

Broad evolutionary differences were observed in our integrated BLA excitatory neuronal datasets compared to the conserved subnuclei enriched by inhibitory neurons. Here, *LAMP5*^+^ and *SATB2*^+^ excitatory neurons were much more abundant in the human BLA (Fig. 4e, f). *LAMP5*^+^ excitatory neurons have also been captured in the human neocortex^45^. Furthermore, as a marker of superficial layer excitatory neurons, *SATB2* is also widely expressed in the neocortex^57^. Interestingly, the *LAMP5*^+^ and *SATB2*^+^ excitatory neurons showed high expression of the lateral and dorsal pallial marker *EMX1* but not of the ventral pallial marker *LHX9* (Fig. 4g). To confirm the presence and spatial distribution of *LAMP5*^+^ and *SATB2*^+^ excitatory neurons in the amygdala, we performed RNAscope mFISH analysis. Results showed that a large number of *SATB2*^+^ neurons resided in the LA portion of the human BLA, but few *Satb2*^+^ neurons were observed in the mouse LA (Fig. 4h, i), consistent with our integrated sequencing data showing that SATB2-2 neurons were mainly derived from humans (Fig. 4f). On the other hand, *LAMP5*^+^ excitatory neurons (*SCL17A7*^+^) were widely distributed in both the human and mouse BLA, including the LA, BA, and BM subregions. In humans, however, the proportion of excitatory neurons expressing *LAMP5* was higher in the LA, but similar in the BA and BM (Fig. 4j, k; Supplementary Fig. S8). These results highlight the differences in the LA across mammals, consistent with the evolutionary increase in LA neuronal populations in humans^20^. Thus, we hypothesize that increased *LAMP5*^+^ and *SATB2*^+^ excitatory neurons in the human BLA, especially the LA, may be due to the substantial increase in the size of the neocortex in the human brain as well as the increase in lateral or dorsal pallial-derived excitatory neuronal (*EMX1* positive) migration to the amygdala.

To explore the possible physiological functions of *SATB2*^*+*^ and *LAMP5*^*+*^ excitatory neurons in the human BLA, we examined differences in gene expression levels across species in these cell types. First, we found that many Patterns in CoGAPS analysis were strongly correlated with *LAMP5* or *SATB2* cell types in the integrated datasets (Supplementary Fig. S7h, i). Next, we detected the expression patterns of these Pattern genes, many of which were highly and specifically expressed in human cells due to the dominance of human cells in these integrated cell types. Interestingly, many Pattern genes correlated with the two neuronal clusters were associated with psychiatric disorders (Fig. 4l). For example, *ADAMTSL3* and *VAV3* are considered candidate genes for schizophrenia^58,59^, and *LTBP1* and *FKBP5* are associated with depressive features in humans^60,61^. The high expression of genes associated with psychiatric disorders implies the potential role of these two clusters in emotional processing in humans. Moreover, we checked the significant biological enrichment GO terms for pattern genes in C0 (*LAMP5*) and C8 (*SATB2-2*). As expected, some of the significant GO terms were related to learning or memory, cognition, and long-term depression (Supplementary Fig. S7j, k), suggesting these human-dominant clusters were relevant to the human superior intelligence such as cognition and learning.

### Diversity and evolution of chicken amygdala cell types

**Fig. 5:**
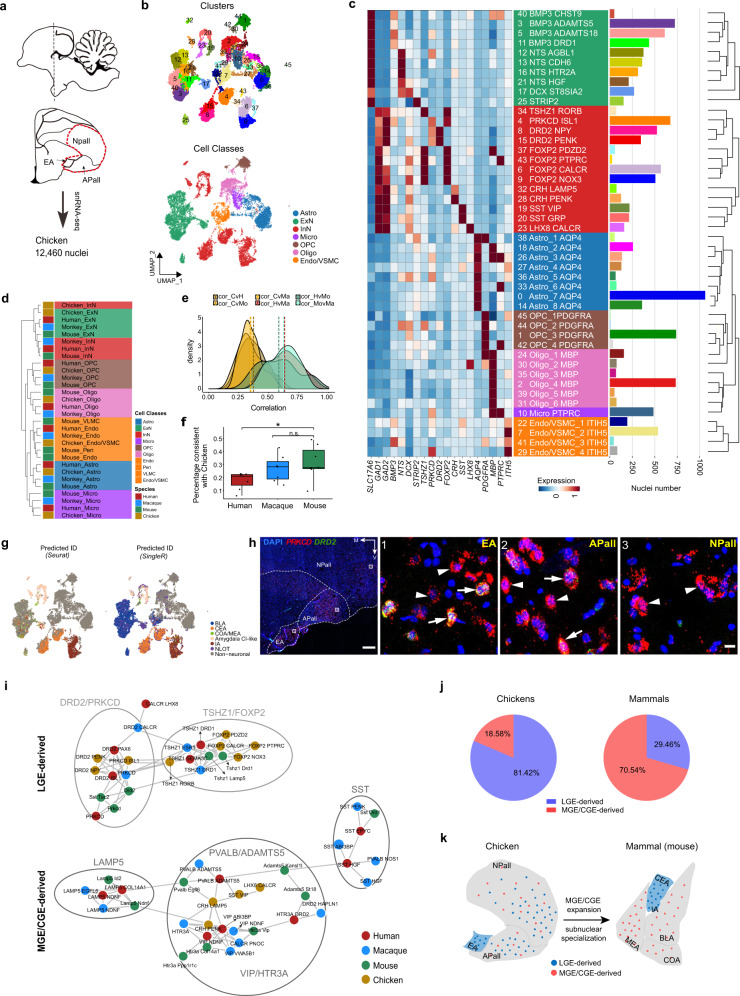
Broad distribution of LGE-derived inhibitory neurons in pallial amygdala in chicken. **a** Workflow for chicken snRNA-seq. Arcopallium (Apall), majority of caudal nidopallium (Npall), and extended amygdala (EA) of chicken were dissected for snRNA-seq. **b** UMAP embedding of chicken datasets, with nuclei colored by cluster (above) and major cell class (below). Endothelial cells, VSMC, and pericytes were collectively referred to as “Endo/VSMC” due to similar transcriptional characteristics. Astro astrocytes; Endo endothelial cells; ExN excitatory neurons; InN inhibitory neurons; Micro microglia; Oligo oligodendrocytes; OPC oligodendrocyte precursor cells; VSMC vascular smooth muscle cells. **c** Taxonomy of 23 neuronal and 23 non-neuronal clusters based on median cluster expression of HVGs (by *hclust* function). Leaves are labeled with cluster names and colored by cell class as in **b**. Heat map showing median expression of cluster signature genes. Bar plot represents the number of nuclei in each cluster. **d** Unsupervised hierarchical clustering of cell classes from different species based on AUROC scores reported by MetaNeighbor. **e** Density histograms showing Pearson correlation distribution of HVGs between pairs of species. Yellow, chicken-mammal pairs; Red, human-macaque pair; Green, primate-mouse pairs. **f** Box plot showing percentage of DEGs in human, macaque, and mouse cell classes consistent with chicken pattern. Center line, box bounds, and whiskers represent mean, 25th to 75th percentile range, and minimum to maximum range, respectively. Dots represent proportion of consistent DEGs in each pair of cell classes. Adjusted *P*-value was calculated by the Wilcoxon test, two-side comparison (* adjusted *P-*value < 0.05). **g** UMAP embedding of chicken datasets, with nuclei colored by predicted cell group ID from Seurat and SingleR methods. **h** mFISH analysis of CEA marker genes *PRKCD* and *DRD2* in chicken amygdala. Example coronal section is shown, scale bar: 500 μm. Boxed regions are enlarged at right. Arrows indicate *PRKCD*^+^ *DRD2*^+^ cells, arrowheads indicate *PRKCD*^+^ *DRD2*^−^ cells, scale bar: 10 μm. Most *PRKCD*^+^ neurons in Npall were *DRD2*^−^. **i** Network plots of inhibitory neurons from four species. The connection edges are displayed between two cell types when the AUROC score is greater than 0.8 (estimated by MetaNeighbor). LGE and MGE/CGE-derived neurons are clustered separately. Major cell types are circled and labeled. **j** Percentage of nuclei derived from LGE or MGE/CGE in chicken and mammals. **k** Schematic of proposed model of MGE/CGE expansion and subnuclear specialization during evolution. See also Supplementary Figs. S9 and S10.

The mammalian amygdala is generally conserved in structure and cellular composition. However, the evolutionary conservation and differences between the mammalian amygdala and that of the evolutionarily distant sauropsids are still unclear. The precise identification of amygdala subnuclei in sauropsids remains controversial due to the obvious differences in brain organization between mammals and sauropsids. Recent studies suggest that the caudal part of the dorsal ventricular ridge (DVR), which is present in all sauropsids, and the surrounding subpallial regions are homologous counterparts of the mammalian amygdala^17,62,63^. To characterize sauropsid amygdala homologs at the single-cell level and compare evolutionary conservation and divergence with the mammalian amygdala, we dissected the chicken caudal DVR (including the arcopallium (APall) and caudal nidopallium (NPall)) and extended amygdala (EA) for snRNA-seq (Fig. 5a; Supplementary Fig. S9a)^64^. Seven classes and 46 cell types were defined in the chicken amygdala homologs (Fig. 5b, c; Supplementary Fig. S9b, c). Hierarchical clustering revealed general conservation of cell classes across species (Fig. 5d). However, obvious differences were observed when all cell types from the mouse and chicken datasets were analyzed, reflected in the tendency of cell types derived from the same species to cluster on the same hierarchical tree branch (Supplementary Fig. S9d). We then performed cross-species expression correlation analysis and found that expression was more consistent between mammals than between mammals and chickens (Fig. 5e), emphasizing the differences among animal classes. On the other hand, chicken cell types showed more similarity with mice than with humans and macaques (Fig. 5f).

We also explored the possible subnuclear origin of chicken cell types based on transcriptomic similarity. Seurat and SingleR methods were applied, showing consistent subnuclear origin predictions for inhibitory neurons, but relatively divergent predictions for excitatory neurons (Fig. 5g). Remarkably, the assumed CEA and IA chicken cell types expressed similar marker genes as those in mice. For example, *DRD2* and *PRKCD* were highly expressed in predicted CEA cells and *TSHZ1* was highly expressed in predicted IA cells. In addition, the *LHX8 CALCR* cluster likely originated from the MEA of chicken amygdala (Fig. 5c, g). These results strongly suggest that inhibitory neuron-enriched subnuclei are highly conserved in amniotes. Recent genetic and migration studies have ascertained that the EA in chickens corresponds to the capsular part of the CEA in mammals^65,66^. To verify this, we detected *PRKCD* and *DRD2* expression patterns in chicken amygdala homologs. The mFISH analysis results showed that numerous *PRKCD*^+^ *DRD2*^+^ and *PRKCD*^+^ *DRD2*^−^ neurons resided in the EA area, consistent with the expression patterns in our snRNA-seq data (Fig. 5h). Our findings confirmed that the EA is at least part of the CEA at the single-cell level and contains cell types with similar gene expression patterns as mammals. However, *PRKCD*^*+*^ neurons were not only located in the EA, but also broadly distributed in the APall and NPall (Fig. 5h). Given that the *PRKCD* and *DRD2* clusters were LGE-derived neurons showing high expression of LGE marker genes (Supplementary Fig. S9e), this observation is consistent with recent research showing that LGE-derived interneurons are broadly distributed in bird brains^62^. Furthermore, our results showed that chickens contained more LGE-derived neurons, while mammals possessed more MGE/CGE-derived neurons in the amygdala (Fig. 5i, j). Based on the above observations, we hypothesize that amygdala subnuclear specialization in chickens may be incomplete due to the widespread distribution of LGE-derived interneurons. On the other hand, the dramatic increase in MGE/CGE-derived neurons in mammals may be a driving force for specialization of amygdala subnuclei, replacing the function of LGE-derived neurons in the pallial amygdala during evolution (Fig. 5k). Although the distribution of *PRKCD* and *DRD2* neurons differed between mammals and chickens, we found that chicken *PRKCD* and *DRD2* neurons also expressed most genes that were conserved in corresponding mammalian clusters (Fig. 3c; Supplementary Fig. S9f and Table S4). This suggests that *PRKCD* and *DRD2* neurons are well-conserved at the gene expression level, even in distantly related species such as the chicken. To explore possible functional differences in these neurons between mammals and chickens, we compared all DEGs and found many genes that were up- and down-regulated in mammalian cells (Supplementary Fig. S9g and Table S8). Interestingly, many of the up-regulated genes are involved in mental conditions and higher cognitive functions, including the schizophrenia- and autism-associated gene *SYN2* and cognition- and intelligence-associated genes *FMN2*, *CTNND2*, and *CACNA1A* (Supplementary Fig. S9g)^67–71^. These up-regulated genes imply that, due to the abundance of MGE/CGE-derived neurons in the mammalian amygdala, LGE-derived PRKCD and DRD2 neurons may be confined to the CEA region and specialized for new functions such as higher cognition and complex emotional regulation in mammals.

To explore potential subnuclear origins of chicken excitatory neurons, correlation analysis between expression profiles was performed, showing no perfect one-to-one correspondence between the mouse and chicken excitatory neurons. However, three populations were clearly visible (Supplementary Fig. S10a). The first corresponded to mouse clusters with high *SLC17A7* expression, the second corresponded to mouse clusters with high *SLC17A6* expression, and the third corresponded to mouse *NLOT* neurons with high *STRIP2* expression. *SLC17A7*^*+*^ neurons were mainly located in the BLA and *SLC17A6*^*+*^ neurons were mainly located in the LA, BM, COA, and MEA, corresponding to lateral or dorsal pallial-derived neurons and ventral pallial-derived neurons, respectively^14,17^. Chicken excitatory neurons were primarily from the caudal DVR, traditionally considered as the ventral pallium in chickens. These results suggest that the caudal DVR may contain many lateral or dorsal pallial-derived neurons, or that convergent evolution may have occurred in the chicken DVR.

Another striking difference between the chickens and mammals was the extreme diversity of non-neuronal cell types in chickens (Fig. 5c; Supplementary Fig. S10b). We captured many rare non-neuronal cell types not captured in mammals, including astrocyte progenitors and developing oligodendrocytes (Supplementary Fig. S10c–f). High non-neuronal activity in the chicken amygdala may be important for neuroplasticity in behavior, but its physiological significance should be further explored.

## Discussion

The amygdala is of particular interest in comparative analysis given its involvement in emotional processing, higher cognitive functions, and various mental disorders^2,72,73^. Here, we established a comprehensive single-nucleus transcriptomic atlas of the amygdala in humans, macaques, mice, and chickens. Our data revealed general evolutionary conservation of the amygdala across species. The CEA and IA were particularly conserved in mammals, with similar structures and cell types even identified in the chicken. The BLA and COA/MEA were relatively divergent during evolution. We also identified several primate-dominant and human-dominant cell types. *LAMP5*^+^ interneurons were much more abundant in primates, and *DRD2*^+^ inhibitory neurons and *LAMP5*^+^/*SATB2*^+^ excitatory neurons were enriched in the human CEA and BLA, respectively.

Inhibitory neuronal cell types were much more conserved than excitatory neuronal cell types in the datasets, consistent with that found in other brain regions^23,62^. Many clusters of inhibitory neuronal cells were matched one-to-one between species. The IA showed the highest conservation, and all mammals contained two types of *TSHZ1*^+^ neurons, i.e., *DRD1*^+^ and *DRD1*^−^. IA neurons are densely innervated by dopaminergic afferents and have been implicated in fear and anxiety behaviors^46^. Recent studies have shown that distinct clusters of intercalated neurons exist in the IA, which exert diametrically opposed roles through mutual synaptic inhibition and access functionally distinct downstream targets^48,74^, similar to fear on and fear off neurons in the CEA^33,75^. Thus, it would be interesting to investigate whether functionally distinct clusters of intercalated neurons correspond to two types of IA neurons in our transcriptomic data in the future. Notably, our data showed that chickens contained numerous *TSHZ1*^+^ clusters with high *FOXP2* expression. The Seurat and SingleR algorithm predicted one *TSHZ1*^+^ cluster as CEA neurons and four clusters as IA neurons. Due to the high expression of *FOXP2* in the avian basal ganglia^76^, we could not rule out the possibility that these *TSHZ1*^+^ clusters were of basal ganglia origin, and thus deeper characterization is required. In addition to *TSHZ1*^+^ clusters, the *DRD2*^+^ clusters also showed conservation across species, although were much more abundant in the human amygdala. Remarkably, the *DRD2*^+^ neurons could also be subclustered into two subtypes, which may have originated from different parts of the LGE based on *PAX6* and *ISL1* expression (Supplementary Fig. S6d)^14^. However, further studies are required to confirm this hypothesis and determine the specific role of each subtype.

Our results support LGE-derived CEA and IA as the most ancient and conserved subnuclei in the amygdala^14,17^. Despite their strong conservation, species–specific specializations were also observed. The mouse CEA contained a cluster, which we named Isl1, that was not captured in humans or macaques. Considering the abundance of the cluster in mice, this result is unlikely to be a sampling issue. In addition to the CEA, mice showed higher cell-type diversity in the COA/MEA, which receive olfactory and vomeronasal inputs, consistent with the relatively larger volume of the COA/MEA and stronger olfactory/vomeronasal system in rodents^21^. These results reflect species-specific adaptations to the environment. The reduction in relative volume and cell-type diversity in the primate COA/MEA suggests cell-type loss during evolution.

Our results showed that the BLA contained most of the excitatory neurons in the amygdala and exhibited extensive evolutionary differences across species, including significantly higher number and diversity of BLA excitatory neuronal clusters in primates. The BLA is part of the pallial amygdala and is proposed to originate from the ventral and lateral pallial sectors based on transcription factor expression and fate mapping^77^. Recent evidence suggests that the dorsal pallium, which contains the neocortex in mammals, also contributes to the cellular composition of the amygdala^78^. Notably, in our study, the *SATB2*^+^ and *LAMP5*^+^ excitatory clusters, which were dominant in the human BLA, demonstrated high expression of the dorsal and lateral pallium marker *EMX1. SATB2* is also a marker of neocortical excitatory neurons^57^, and *LAMP5*^+^ excitatory neuronal clusters are reported in the human neocortex^26^. Thus, we propose that more and more neocortical-derived neurons migrated to the amygdala following expansion of the neocortex during higher primate evolution, and this evolutionary gain may play an important role in the integration of signal inputs and higher cognitive functions.

Although abundant cell types were identified in the amygdala of humans, macaques, mice, and chickens, we anticipate that more cell types will be found with deeper sampling, especially in humans and macaques, given the absolute volume of the amygdala across species. Indeed, we identified a *Sst*^+^ cluster in the mouse CEA through snRNA-seq profiling of the CEA directly (Supplementary Fig. S4a, b), which may correspond to previously reported *Sst*^+^ neurons in the mouse CEA^34^. However, no corresponding SST^+^ neurons from the CEA were captured in the human or macaque datasets, indicating that capture of rare cell types may require additional sampling. Moreover, the CEA *Sst*^+^ cluster could be segregated into three subclusters (Supplementary Fig. S11), indicating that cell types in our datasets could be further subclustered and more cell types could be identified.

Single-cell transcriptome analysis, especially in cross-species analysis, relies on efficient data integration. In our study, we used the BBKNN algorithm method, which can effectively combat intra-species batch effects, as shown in Supplementary Fig. S1b. In the process of integrating datasets across species, we chose widely used Seurat workflow^79^ with some adjustments. As shown in Supplementary Fig. S5a and Fig. 4a, most cells were well mixed across species except for a few species-dominant cell types. However, different datasets may require different batch-corrected methods, since too strong batch-corrected methods may eliminate biologically meaningful inter-/intra-species differences. Extensive comparison of the impact of different batch-corrected methods on the integrated datasets will facilitate the discovery of more intra-species cell types and more conserved or species-specific cell types across species in the future.

## Materials and methods

### Sample collection and ethical compliance

Postmortem adult human brain tissue samples were obtained from the National Human Brain Bank for Health and Disease in Hangzhou (NHBBHD), China. Written informed consent for the use of the tissues in research was obtained from donors or their families. All postmortem brain samples were from donors with no obvious brain disease based on pathological diagnosis by the NHBBHD. Postmortem tissue collection was approved by the ethics committees of NHBBHD and Zhejiang University. Three fresh human amygdala tissues (Supplementary Table S1) were collected for snRNA-seq, and one 10% formalin-fixed human amygdala tissue sample was collected for RNAscope multiplex fluorescence in situ hybridization (mFISH).

Rhesus macaque (*Macaca mulatta*) experiments were conducted in accordance with the Principles for the Ethical Treatment of Non-Human Primates and were approved by the Institutional Animal Care and Use Committee of the Institute of Biophysics, Chinese Academy of Sciences, and the Animal Advisory Committee at Zhejiang University. For snRNA-seq, three adult and senile macaques (Supplementary Table S1) were deeply anesthetized with ketamine (10 mg/kg) and perfused with cold artificial cerebrospinal fluid (ACSF) after the pedal withdrawal reflex was eliminated. Whole brains were rapidly extracted and placed on a precooled metal plate. The macaque amygdala was dissected within 30 min according to anatomical landmarks, such as optic tract, hippocampus, and ventrolateral temporal neocortex. Fresh tissues were snap-frozen in liquid nitrogen and stored at −80 °C until use.

All domestic chicken (*Gallus gallus*) experiments were reviewed and approved by the Animal Advisory Committee at Zhejiang University. For snRNA-seq, two adult chickens (Supplementary Table S1) were sacrificed by CO_2_ asphyxiation, and whole brains were rapidly extracted into oxygenated ice-cold sucrose-based ACSF containing: 25 mM sucrose, 10 mM D-glucose, 24 mM NaHCO_3_, 3 mM KCl, 5 mM MgCl_2_, 2 mM CaCl_2_, 1.25 mM NaH_2_PO_4_, and 2 mM MgSO_4_. Coronal slice sectioning was performed as reported previously^80^. Briefly, the forebrain was mounted on a steel plate, and 300-μm-thick coronal sections were cut in the cold, sucrose-based ACSF with a vibratome slicer (VT1200s, Leica Biosystems, Nussloch, Germany). Carbogen (95% O_2_, 5% CO_2_) was bubbled continuously during sectioning. Amygdala homologs in the caudal ventrolateral part of the forebrain, including the arcopallium, most of the caudal nidopallium, and extended amygdala, were dissected under a dissection microscope (SMZ645, Nikon). Dissected tissue was snap-frozen in liquid nitrogen and stored at −80 °C before further processing. For RNAscope mFISH analysis, one adult male chicken was sacrificed by CO_2_ asphyxiation, and whole brains were rapidly extracted into 4% paraformaldehyde (PFA) for fixation.

All procedures involving C57BL/6 J mice (Jackson Labs, #000664) were approved by the Animal Advisory Committee at Zhejiang University. For snRNA-seq, eight-week-old male and female mice (Supplementary Table S1) were deeply anesthetized with 1% sodium pentobarbital (0.1 g/kg body weight) and transcardially perfused with ice-cold oxygenated N-methyl-D-glucamine (NMDG)-based ACSF containing: 93 mM NMDG, 2.5 mM KCl, 1.25 mM NaH_2_PO_4_, 30 mM NaHCO_3_, 20 mM HEPES, 25 mM glucose, 5 mM sodium ascorbate, 2 mM thiourea, 3 mM sodium pyruvate, 10 mM MgSO_4_.7H_2_O, and 0.5 mM CaCl_2_.2H_2_O. Mice were rapidly decapitated and whole brains were extracted into ice-cold oxygenated NMDG-based ACSF, with 300-μm-thick coronal sections then cut in the cold, NMDG-based ACSF with a vibratome slicer (VT1200s, Leica Biosystems). Carbogen (95% O_2_, 5% CO_2_) was bubbled continuously during sectioning. The whole amygdala, basolateral amygdalar complex (BLA), and central amygdalar nucleus (CEA) were rapidly dissected under a dissection microscope from different sections, snap-frozen in liquid nitrogen, and stored at −80 °C. For RNAscope mFISH, adult male C57BL/6 J mice were anesthetized with 1% sodium pentobarbital (0.1 g/kg body weight) and transcardially perfused with 1× phosphate-buffered saline (PBS), followed by 4% PFA. Brains were extracted into 4% PFA for post fixation.

### Isolation and purification of nuclei

For human specimens, frozen tissues were placed on a precooled metal plate and cut into 1–2-mm-thick coronal sections using a cold scalpel according to spatial coordinates. For donor CBB052, each section was used for nuclei isolation. For donors CBB037 and CBB038, only one typical intermediate coronal section was used for nuclei isolation. Entire macaque amygdala samples and dissected mouse and chicken samples were utilized for nuclei isolation. Nuclei were isolated and purified following the 10× Genomics protocols (CG000393 • Rev A), with some modification. Briefly, the frozen tissues were homogenized on ice in lysis buffer containing: 10 mM Tris-HCl, 10 mM NaCl, 3 mM MgCl_2_, 0.01% NP-40 (ThermoFisher), 1 mM β-mercaptoethanol, and 0.2 U/μL RNase inhibitor (Promega) by a glass homogenizer. Tissues were dounced until there were no visible clumps. Homogenates were incubated on ice for 5 min, and an equal volume of HEB medium (Hibernate E/B27/GlutaMAX, Gibco) was added to stop lysis. Cell debris and large clumps were removed using 30-μm (mouse and chicken) and 40-μm (human and macaque) filters (pluriSelect), and the supernatant was centrifuged at 500× *g* and 4 °C for 5 min. To further remove debris, Myelin Removal Beads II (Miltenyi Biotec, mouse tissues) and Debris Removal Solution (Miltenyi Biotec, human, macaque, and chicken tissues) were applied according to the manufacturer’s instructions. Nuclei were washed 1–2 times and resuspended in wash and resuspension buffer containing 1× PBS, 1% bovine serum albumin (BSA, Sigma), and 0.2 U/μL RNase inhibitor, then collected by centrifugation at 500× *g* and 4 °C for 5 min. DAPI DNA dye (ThermoFisher) was added and nuclei were counted manually under a fluorescence microscope (BX53, Olympus). The nuclear suspension was diluted to a concentration of 500–1000 nuclei/μL for loading onto the 10× Chromium instrument.

### SnRNA-seq library preparation and sequencing

Nuclear suspension loading onto the Chromium Next GEM Chip, nuclear barcoding, cDNA amplification, and library construction were carried out according to the 10× Genomics protocols (CG000204 Rev D). Libraries were sequenced on the Illumina NovaSeq 6000 System at LC-Bio Technology Co., Ltd. in Hangzhou, China.

### SnRNA-seq data processing

All raw snRNA-seq data are accessible from the Gene Expression Omnibus database (GEO: GSE195445). The snRNA-seq data constructed using 10× Genomics were processed through the Cell Ranger Single-Cell Software Suite (v3.1.0)^81^, which performed sequencing read alignment and gene expression quantification. The sequencing reads from humans were aligned to the GRCh38 human reference genome, while the mouse, macaque, and chicken sequencing reads were aligned to the mm10-3.0.0, Mmul_10.102, and GRCg6a species reference genomes, respectively. We used the *CellRanger aggr* pipeline to merge cells from the same species under different samples, with the parameter “*normalize* = *none*”. We applied quality control procedures to cells in each species step by step, first computing the highest density interval for the number of genes detected (“n_genes_by_counts”) and the percentage of mitochondrial genes expressed (“pct_counts_mt”) using the *hdi* function in the HDInterval R package with the parameter “*credMass* = *0.98*”. Cells outside the confidence interval were filtered. We then removed potential doublets reported by Scrublet^82^, with default parameters. After strict quality control analysis, we retained 91,699 nuclei with a median of 9417 unique molecular identifiers (UMIs) and 3617 genes in the human dataset; 45,626 nuclei with a median of 4631 UMIs and 2373 genes in the macaque dataset; 54,186 nuclei with a median of 5020 UMIs and 2316 genes in the mouse dataset; and 12,460 nuclei with a median of 3660 UMIs and 1716 genes in the chicken dataset. We then used the *normalize_total* and *log1p* functions in SCANPY (v1.8.2)^83^ to normalize (library-size correct) and logarithmize the raw count matrix with default parameters. Next, we detected highly variable genes (HVGs) using the *scanpy.pp.highly_varible_genes* function with the parameters “*min_mean* = *0.0125, max_mean* = *3, min_disp* = *0.5*” and selected all HVGs for downstream analysis. Finally, we used the *scanpy.pp.regress_out* function to regress the effects of total counts and mitochondrial gene expression percentage.

### Dimensionality reduction and unsupervised clustering of single-species datasets

We employed the SCANPY^83^ workflow to reduce dimensionality and embed the neighborhood graph on the gene expression matrix. We first performed principal component analysis (PCA) using the *scanpy.tl.pca (svd_solver* = *‘arpack’, n_comps* = *100)* function to obtain a PC matrix with 100 components. We then used the *bbknn* function with the parameters “*batch_key* = *‘sample’, n_pcs* = *75*” to align the batch effects from samples and computed the neighborhood graph. For embedding the graph in two dimensions, we performed UMAP analysis using the *scanpy.tl.umap* function. Unsupervised clustering was realized using the *sc.tl.leiden* function with appropriate *resolution* parameters adapted to the diverse datasets. The parameters were set to: “*resolution* = *1.5*” for mice, “*resolution* = *1.5*” for humans, “*resolution* = *2.8*” for macaques, and “*resolution* = *2.2*” for chickens. Cluster-specific DEGs were identified using the *scanpy.tl.rank_genes_groups* function with the Wilcoxon test. For clarity, we unified the naming of cell populations in the diverse conditions as follows: clusters from unsupervised clustering were named “Cell Clusters”, cell populations containing multiple clusters were named “Cell Classes”, and amygdala subnuclear distributions were named “Cell Groups”. Cell classes in each species dataset were annotated using canonical markers of cell types, as shown in Fig. 1c.

### Integrating snRNA-seq datasets across species

To identify homologous cell types across species, we performed three rounds of dataset integration: Mammal Datasets (all nuclei from mammals), Inhibitory Neuronal Datasets (inhibitory neurons from mammals), and BLA Excitatory Neuronal Datasets (nuclei from BLA cell group in mammals). We employed the Seurat (v4.0.5)^79^ workflow to integrate datasets across diverse species. The vignette of SeuratDisk (https://github.com/mojaveazure/seurat-disk) was used to convert AnnData objects to Seurat objects. The BiomaRt^84^ package was applied to reduce raw matrices to only orthologous genes (13,478 genes) with one-to-one orthologues defined in humans, macaques, and mice. The *SelectIntegrationFeatures* function in Seurat was used to select repeatedly variable features across datasets. We then applied the *FindIntegrationAnchors* function to identify cross-dataset pairs of anchors, and the *IntegrateData* function was used to create an integrated assay using pre-computed anchors. The integration procedures used default parameters, except “dim=1:50”. The integrated matrix (batch-corrected) was used for dimensionality reduction and unsupervised clustering with the *RunUMAP* and *FindClusters* functions, respectively. We set parameters to “*resolution* = *0.35”* and “*resolution* = *0.25”* when the *FindClusters* function was used for the Inhibitory Neuronal Datasets and BLA Excitatory Neuronal Datasets, respectively. For Mammal Datasets and BLA Excitatory Neuronal Datasets integration, each Seurat object from different species was normalized using the *SCTransform* function in Seurat. To integrate the Inhibitory Neuronal Datasets, we combined the top 100 DEGs from each cluster within each species as features for the *FindIntegrationAnchors* function. All adjustments were attempts to eliminate species-specific batch effects.

### Spatial mapping of amygdala subnuclei

We mapped the mouse snRNA-seq gene expression profile to the mouse ISH atlas of the Allen Institute for Brain Science (http://mouse.brain-map.org) at the cluster level^85^. The ISH data of each slice were summarized into voxel dataset, providing the gene expression profile of each voxel (Gene expression value X voxel). The brain structures file of each slice showed the anatomical annotation of each voxel (voxel X structures). First, we generated whole brain plots with only amygdala subnuclei annotation in each slice using brain structures file to select the proper coronal slice as reference image. We chose slice 37 to plot reference image, where we divided the amygdala into five subnuclei: BLA (Basolateral amygdalar complex), IA (Intercalated amygdalar nucleus), CEA (Central amygdalar nucleus), MEA (Medial amygdalar nucleus), and COA (Cortical amygdalar area). Then, we downloaded the ISH data of slice 37 to map gene expression on the reference image. We calculated the average expression value of the top 5 DEGs in each cluster and then, projected it on the reference image to infer the subnuclei distribution of clusters. (Only DEGs that appeared in the ISH coronal gene expression profile were retained).

### Cell group prediction

#### Seurat label transfer

We first annotated clusters (Cell Group labels) in mice based on ISH data from the Allen Brain Atlas (https://portal.brain-map.org/) and Ro/e (ratio of observed to expected number of nuclei) distribution analysis, as shown in Supplementary Fig. S3. We quantified the fraction of clusters from each amygdala cell group based on transcriptomic similarity with mouse datasets. Seurat supports the projection of a list of reference labels (i.e., Cell Group labels in mice) onto a query object (i.e., human/macaque/chicken objects). We used the *FindIntegrationAnchors* function with the parameter “*dims* = *1:50*” to identify anchors between the reference and query datasets, then used these anchors with the *IntegrateData* function to classify query cells based on the reference labels. We added “predicted.id” to the metadata of the Seurat object for data visualization and showed cell group ID on the UMAP plot. We calculated the proportion of cells from different cell groups in each cluster and annotated clusters with the highest proportion.

#### SingleR analysis

SingleR^86^ is an effective tool to infer the development origin of each cell. Here, we used SingleR to transfer mouse cell group labels to the human/macaque/chicken datasets. We used the *pairwiseTTests* and *getTopMarkers* functions in the R package scran to identify the top markers for each pairwise group of cells^87^. We performed SingleR procedures with default parameters and visualized “predicted.id” on the UMAP plot.

### Cluster dendrograms

For clusters of each species, cluster dendrograms were established based on hierarchical clustering across excitatory neurons, inhibitory neurons, and non-neuronal cells. Average expression of HVGs was calculated for each cluster, and hierarchical clustering was performed using the *hclust* R function. The dendrogram branches were reordered manually without changing the tree structure.

### Cell class DEG expression patterns across species

We selected the top 500 DEGs of each cell class across species. Venn diagrams were generated using the VennDiagram R package to visualize DEG intersections across species for each given Cell Class cluster.

To show the consistency of expression patterns in neurons across species, we combined the top 100 DEGs of excitatory and inhibitory neurons from every two species (human-macaque, human-mouse) and calculated the log_2_FC value for each gene between classes of excitatory and inhibitory neurons. We defined genes with log2FC > 1 in mice or macaques and log2FC > 1 in humans as “ExN_sig”, and log2FC < (−1) in mice or macaques and log2FC < (−1) in humans as “InN_sig”. Expression patterns of neuronal DEGs were presented in a scatter plot.

### MetaNeighbor analysis

To compare the conservation and divergence of co-expression in randomly defined gene sets, we performed unsupervised MetaNeighbor analysis on cross-species integrated datasets^88^. We first used the *get_variable_genes* function to select the top quartile of variable genes. Cross-species comparisons at different levels (Cluster/Cell Group/Cell Class levels) were performed using the *run_MetaNeighbor* function. The output area under the receiver operator characteristic curve (AUROC) scores of each pair of labels was plotted as a heat map. We removed cells from the nucleus of the lateral olfactory tract (NLOT) subnuclei to analyze amygdala cell group characteristics as their definition was not clear enough in the amygdala.

We calculated the total AUROC scores in the box plot using the AUROC scores from the original clusters of all species in Supplementary Fig. S4b, which showed the accumulation of species-dependent expression differences in the amygdala cell groups.

To visualize the relationships between homologous excitatory neuronal clusters across chickens and mice, the AUROC scores of selected clusters were taken and loaded into Cytoscape^89^ to draw a network graph, with the connection edge between two cell-type clusters displayed when AUROC > 0.75. We performed the same analysis on inhibitory neuronal clusters across all four species (humans, macaques, mice, and chickens) and visualized the network using the R package igraph (edges displayed when AUROC > 0.8).

### Calculating DEGs in integrated datasets

To identify conserved and species-enriched DEGs in different cell types across species, we used the *FindAllMarkers* function in Seurat for all cell types in each species. Positive DEGs were reported using the *ROC* test with default parameters. To avoid computational memory issues, we down-sampled the Seurat object to a small number of cells (200 cells/species cell group in the Mammal Datasets and 80 cells/species cluster in the Inhibitory Neuronal Datasets). We first calculated the DEGs of cell types separately in each species. In DEGs of each cell type, conserved DEGs were defined as DEGs present in both species, and species-enriched DEGs were defined as DEGs only expressed in one species. The *DoHeatmap* function in Seurat was used to plot the heat map of DEGs with small volume cells (as mentioned above). When we performed DEG analysis of the Mammal Datasets across cell groups, we removed the “PL (paralaminar nucleus)” and “NLOT” groups as these two group-derived cells were only captured in two species.

### Cell group expression divergence

For each pair of homologous cell groups between humans and macaques or humans and mice, we calculated the average expression of each orthologous gene, with scatter plot visualization after log-transformation. We calculated divergent genes in each cell group between pairs of species (human-mouse/human-macaque) using the limma R package^90^. We identified significant divergent genes based on log_2_FC > 1 and adjusted *P-*value < 0.01 across paired species. Pearson correlations were calculated to evaluate the relationships between humans and macaques or humans and mice using the *cor.test* function. Average expression of all divergent genes was calculated and visualized in a heat map with the parameters “*cluster_columns* = *T, cluster_rows* = *T*”.

### SCENIC analysis

We used the SCENIC^91^ workflow to analyze activated regulons in each amygdala cell group. Raw count matrices from the single species were input to infer a co-expression network using the *GRNBoost2* function. The RcisTarget R package was used to identify transcription factor binding motifs, and the AUCell R package was used to quantify regulon activity in single cells. We used the *wilcox.test* function to determine the differentially activated regulons in each cell group and the *p.adjust* function was used to correct multiple hypotheses. We selected and plotted (heat map) the top five regulons intersecting the three species.

### PAGA developmental trajectory analysis

We reconstructed the PL (paralaminar nucleus) nucleus-derived cell development trajectories using the PAGA package^83^ for the human and macaque datasets. The *scanpy.tl.paga* function was used for PAGA graph computation and non-significant edges were filtered out based on connectivity > 0.25. Edge connectivity between the C36 cluster (in macaques) and C24 cluster (in humans) with all other cluster nodes was visualized in the bar plot. We recomputed embedding based on PAGA initialization and calculated diffusion pseudotime using the *scanpy.tl.dpt* function (root cell set as a cell in macaque C36 cluster or human C24 cluster). We showed the PAGA trajectories using a set of genes.

### Homologous cell types across species

We defined consensus clusters by comparing the overlap of species-original clusters in cell types of integrated Seurat objects (reported by *cca* algorithm) between humans and macaques and between humans and mice, as described previously^45^. For each cluster pair, we calculated overlap values, i.e., the sum of the minimum proportion of nuclei in each original cluster that overlapped with each cell type. Cluster overlap values were scaled to 0–1 and visualized in a heat map.

### Ro/e analysis

To quantify species distribution of clusters, we calculated the ratio of observed to expected number of nuclei (Ro/e) for each cell type cluster across species using the chi-square test^92,93^, with higher Ro/e values indicating a greater degree of enrichment in a species.

We also used Ro/e analysis to quantify cell group distribution of clusters in the Inhibitory Neuronal Datasets, and tissue distribution of clusters in the mouse dataset. The Ro/e ratio for each cluster was calculated across Cell Group or dissection origin.

### Enrichment analysis

We applied the *enricher* function in the clusterProfiler R package^94^ for gene set enrichment analysis. Gene sets of “c5 category” from the MsigDB dataset (http://www.gsea-msigdb.org/gsea/msigdb/collections.jsp) were loaded using the R package *msigdf*. Adjusted *P*-values and gene counts for enriched pathways were plotted (bar plot) and networks of the selected gene sets were visualized using the *cneplot* function in the R package *enrichplot*. To quantify gene set scores across clusters, we calculated the average expression of genes from a given gene set in a single cell.

We used the GSVA R package^95^ to analyze gene set variation and the limma package^90^ to calculate the *T*-value of differentially enriched pathways.

### CoGAPS analysis

We used CoGAPS^96^ to construct a latent space learning graph based on single-cell gene expression matrices, following previous research^97,98^. As a nonnegative matrix factorization (NMF) algorithm, CoGAPS factorizes the gene expression matrix into two matrices: i.e., Pattern (cell × pattern) and Amplitude matrices (genes × pattern). Here, we performed NMF for each species step by step. First, we split the normalized gene expression of each species from integrated analysis and reduced the expression matrix into a matrix containing only 1000 HVGs (reported using the *FindVariableFeatures* function in Seurat). We then used the *CoGAPS* function with the parameters “*nPatterns* = *150, nIterations* = *500, sparseOptimization* = *TRUE, seed* = *77, nSets* = *15*” to obtain a CogapsResult object.

To determine the biological performance of patterns across different species, we used the R package *projectR* (https://github.com/genesofeve/projectR/) to project a new gene expression matrix onto the CoGAPS Pattern matrices. Human CoGAPS amplitude matrices were input as source data, while macaque and mouse gene expression matrices were input as target data. We calculated correlation of the Pattern matrix with the global features matrix (i.e., cluster labels) using the *cor* function and visualized these pattern correlations in a dot plot (for clarity, only patterns with correlation values > 0.1 were plotted). We selected the top 30 most heavily weighted genes from a given pattern and visualized scaled average expression across clusters in each species on the heat map.

### Topic modeling

We used the *FitGoM* function in the CountClust R package to fit latent Dirichlet allocation topic models to the raw count matrix^99,100^. Specifically, we filtered out genes with zero expression from the raw matrix prior to fitting the topic models. We then ran the *FitGoM* function with the parameter *“k* = *25”* to obtain an object containing two matrices: Factors and Loadings. We added the loading weights of each cell to the metadata of the Seurat object and drew the topic loading weights in the UMAP coordinates. Finally, to interpret the biological processes of the selected topics, we used the *diff_count_analysis* function with default parameters to analyze DEGs among topics. We performed enrichment analysis of selected topic DEGs (*P*-value < 0.01, log_2_FC > 2) and gene sets from the MsigDB datasets “c8 category” were used as source data. The log_2_FC values (reported by *volcano_plot* function) of genes from selected pathways were plotted in a bar plot.

### RNAscope mFISH analysis

Formalin-fixed human amygdala tissue and PFA-fixed chicken and mouse brains were embedded in optimal cutting temperature medium (OCT, Sakura), frozen with dry ice, and stored at −80 °C. The frozen tissues were sectioned (15–20 μm) onto Superfrost Plus glass slides (Epredia). Sections were dried at −20 °C and stored at −80 °C until use. RNAscope mFISH experiments were performed as manufacturer’s instruction for fixed-frozen tissue samples (Advanced Cell Diagnostics), but with the following modifications: (1) protease treatment was lengthened to 40 min when using human tissues; (2) TrueBlack (Biotium) was used to quench autofluorescence in human and chicken tissues after the RNAscope experiment. An Olympus FV3000 fluorescent microscope was used for imaging (10×, 20×, and 40× air lenses and 60× oil immersion lens), and image processing was carried out using Image J. Positive cells were counted manually, containing ≥ five RNA spots for that gene. Lipofuscin autofluorescence was distinguished based on the broad fluorescence spectrum of lipofuscin.

### Quantification and statistical analysis

Details of the statistical analyses used and sample size (*n*) for each analysis were present within the figure legends. Statistical significance was determined using GraphPad Prism and R software, unless otherwise noted. For statistical comparisons, *P*-values below 0.05 were considered statistically significant.

## Supplementary information

Supplementary Information

Supplementary Table S1

Supplementary Table S2

Supplementary Table S3

Supplementary Table S4

Supplementary Table S5

Supplementary Table S6

Supplementary Table S7

Supplementary Table S8

## Data Availability

The datasets generated during this study have been deposited in the Gene Expression Omnibus public database under accession number GSE195445.
