# Supplementary material for: Molecular and cellular evolution of the amygdala across species analyzed by single-nucleus transcriptome profiling
Source: Cell Discov. 2023 Feb 14;9:19. doi: 10.1038/s41421-022-00506-y (PMC9929086; doi:10.1038/s41421-022-00506-y)
Supplement: Supplementary file 1 — Supplementary Information [file 41421_2022_506_MOESM1_ESM.pdf]

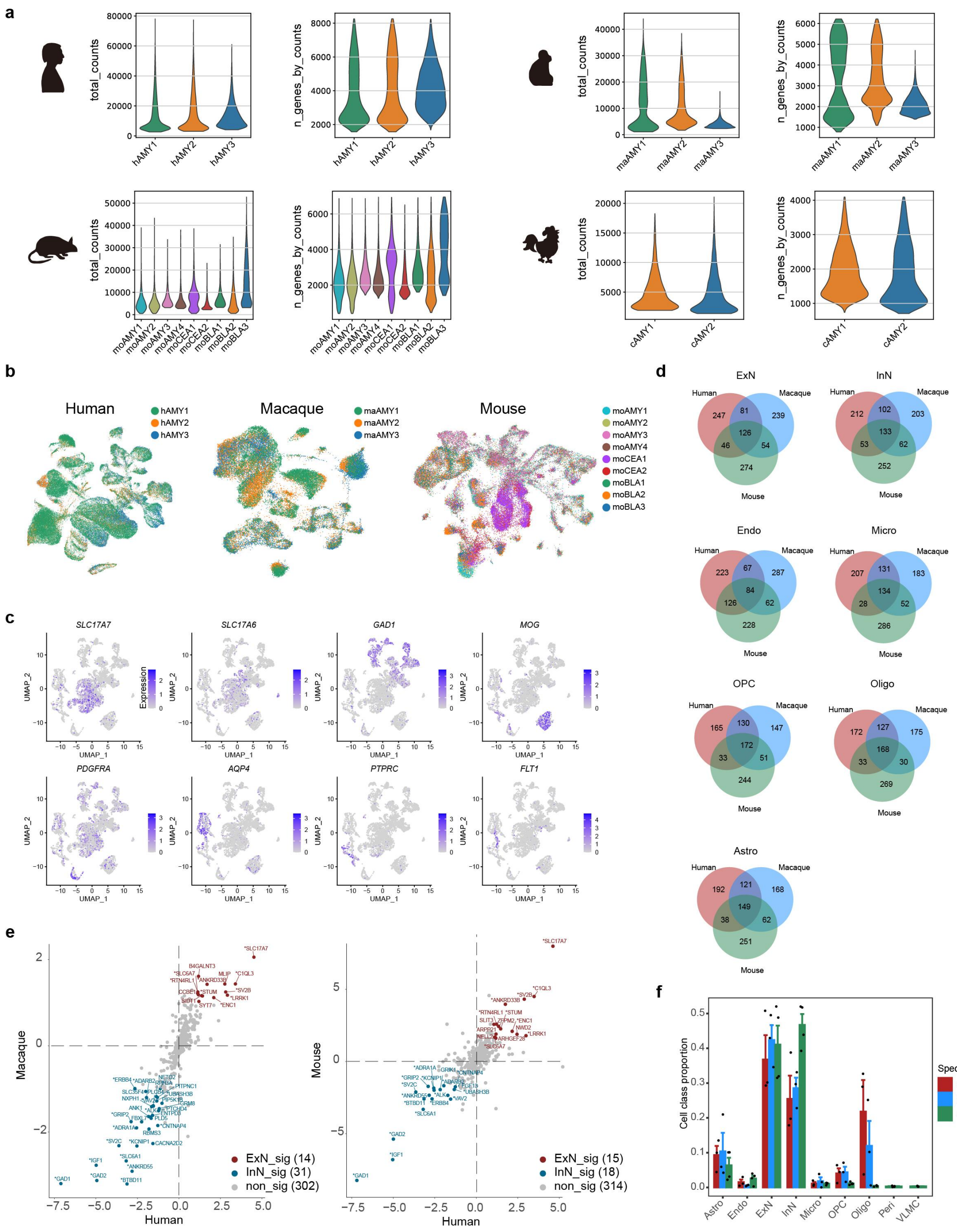

Fig. S1 See next page for caption.

Fig. S1 snRNA-seq quality and integration of human, macaque, and mouse datasets, related to Fig. 1

a Quality control plot of each species dataset. Violin plot displaying total number of unique molecular identifiers (UMIs), detected gene numbers in humans, macaques, mice, and chickens. cAMY: chicken amygdala.

b UMAP plots showing well-mixed nuclei from different samples or donors. Samples and donors are colored as in (**a**).

c Feature plots showing expression of cell class marker genes.

d Venn diagrams displaying DEG numbers of each cell class shared or enriched across species. Top 500 DEGs of each cell class across species were used for analysis.

e log2 fold change (log2FC) of DEGs showing consistent excitatory (red) and inhibitory (blue) neuronal marker genes between species. Dots represent the union of the top 100 DEGs of ExN and InN from human and macaque or human and mouse. Asterisks indicate shared markers across three species. The number of DEGs were labeled in round brackets.

f Relative proportions of nuclei in each cell class across species. Dots represent individual samples. Data are represented as mean ± SEM.

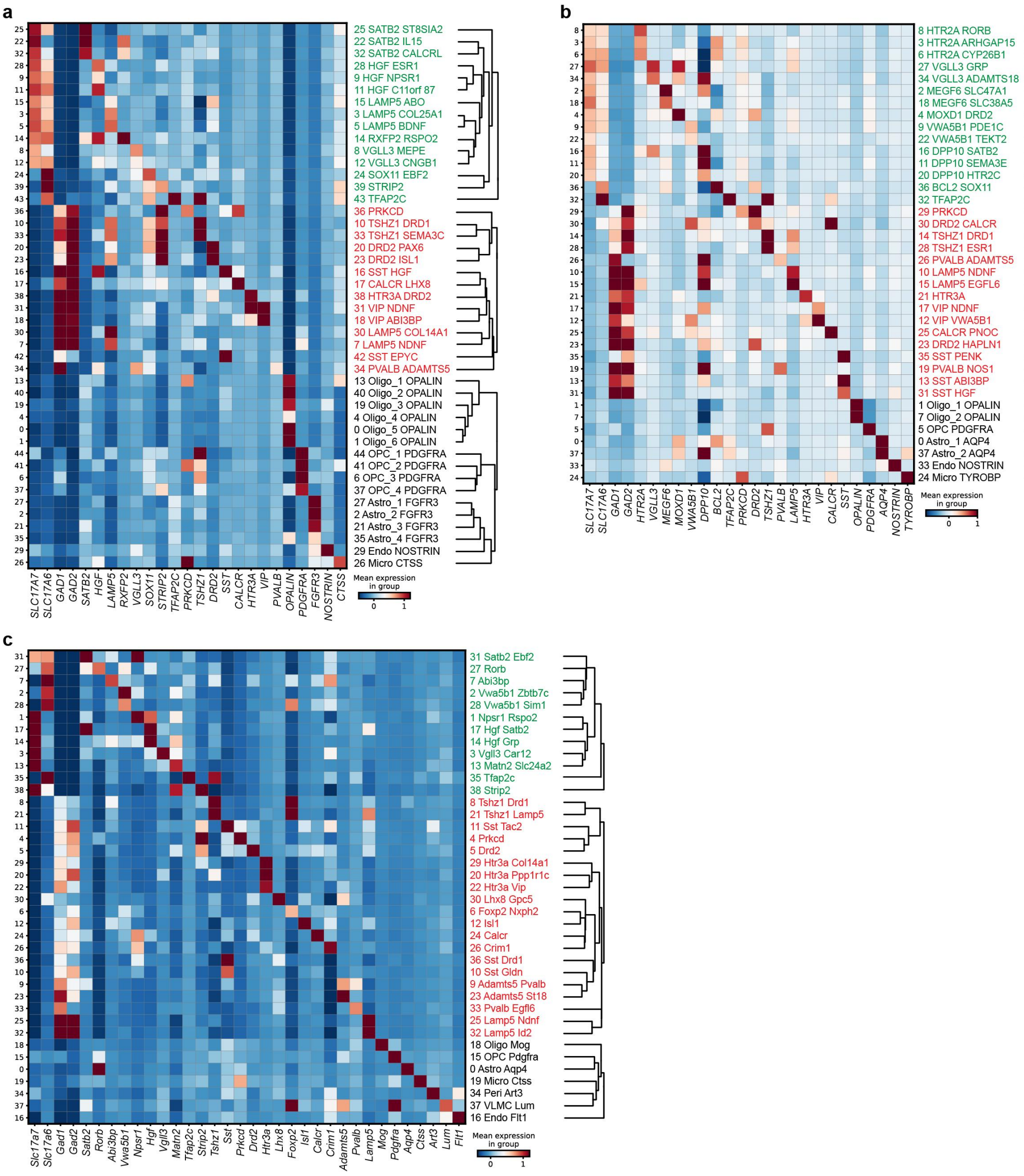

Fig. S2 See next page for caption.

Fig. S2 Taxonomy of cell types in human, macaque, and mouse amygdala, related to Fig. 1

a-c Taxonomy of clusters based on median cluster expression of HVGs. Leaves are labeled with within-species clusters (green, excitatory neurons; red, inhibitory neurons). Heat map showing median expression of cluster signature genes. We defined 15 excitatory neuronal clusters and 14 inhibitory neuronal clusters in human datasets in (**a**); 15 excitatory neuronal clusters and 16 inhibitory neuronal clusters in macaque datasets in (**b**); and 12 excitatory neuronal clusters and 20 inhibitory neuronal clusters in mouse datasets in (**c**). Hierarchical clustering was performed on excitatory neuronal clusters, inhibitory neuronal clusters, and non-neuronal clusters, separately (by *hclust* function).

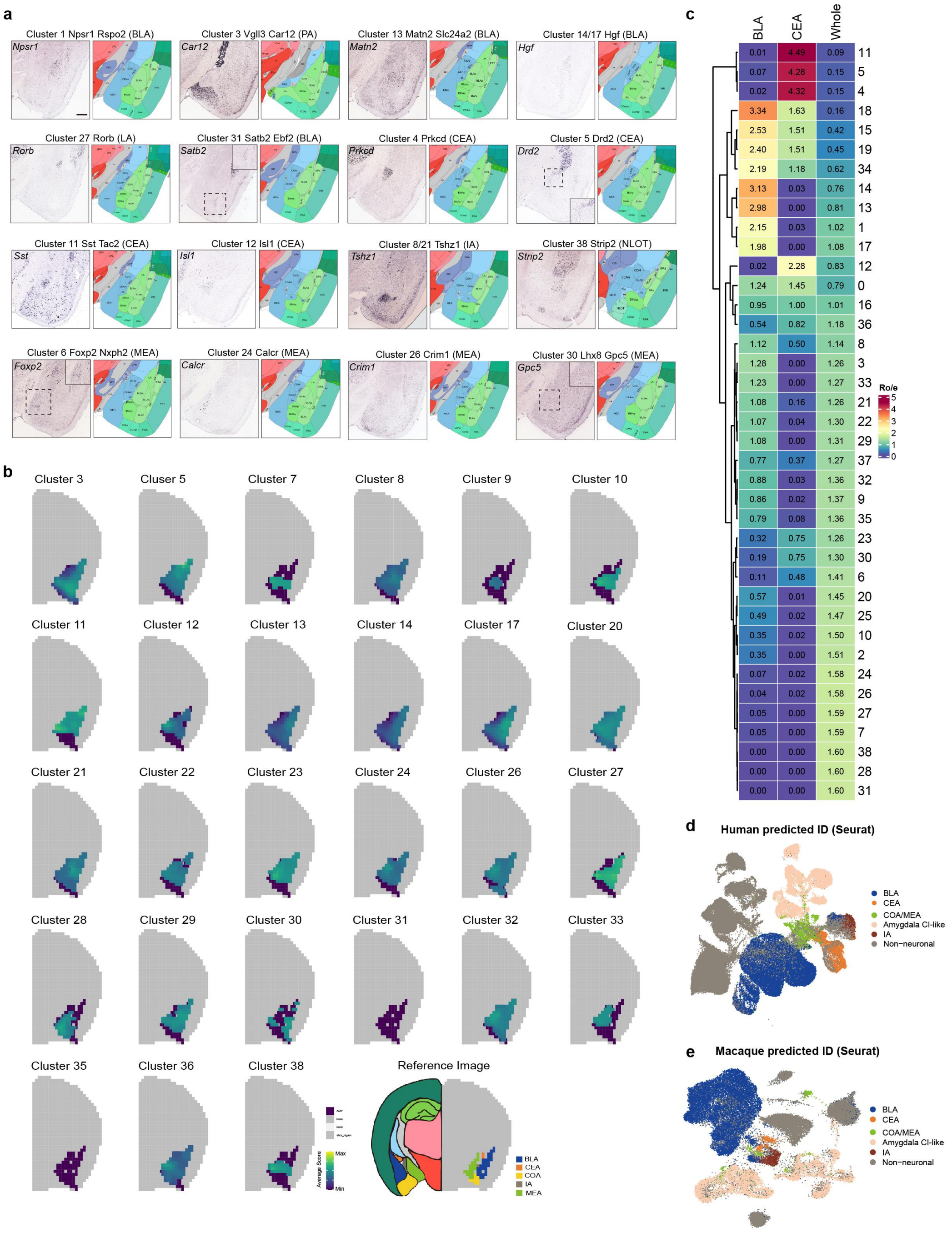

Fig. S3 See next page for caption.

Fig. S3 Cell type distribution in subnuclei of amygdala, related to Fig. 2

a Spatial expression patterns of mouse cell-type marker genes in mouse amygdala, based on RNA *in situ* hybridization (ISH) data from Allen Mouse Brain Atlas, scale bar: 300 μm.

b Spatial expression patterns of top five DEGs in each mouse cluster in amygdala. Left panel image indicates reference coronal section at slice 37 of Allen Brain Reference Atlas. For every selected cluster, average score of top five DEGs is depicted by colormap.

c Ro/e analysis of mouse cell types based on dissection origin. The higher the value of Ro/e, the greater enrichment of cell types in the corresponding dissection area.

d, e UMAP plots of human (**d**) and macaque (**e**) datasets, with nuclei colored by predicted ID from Seurat.

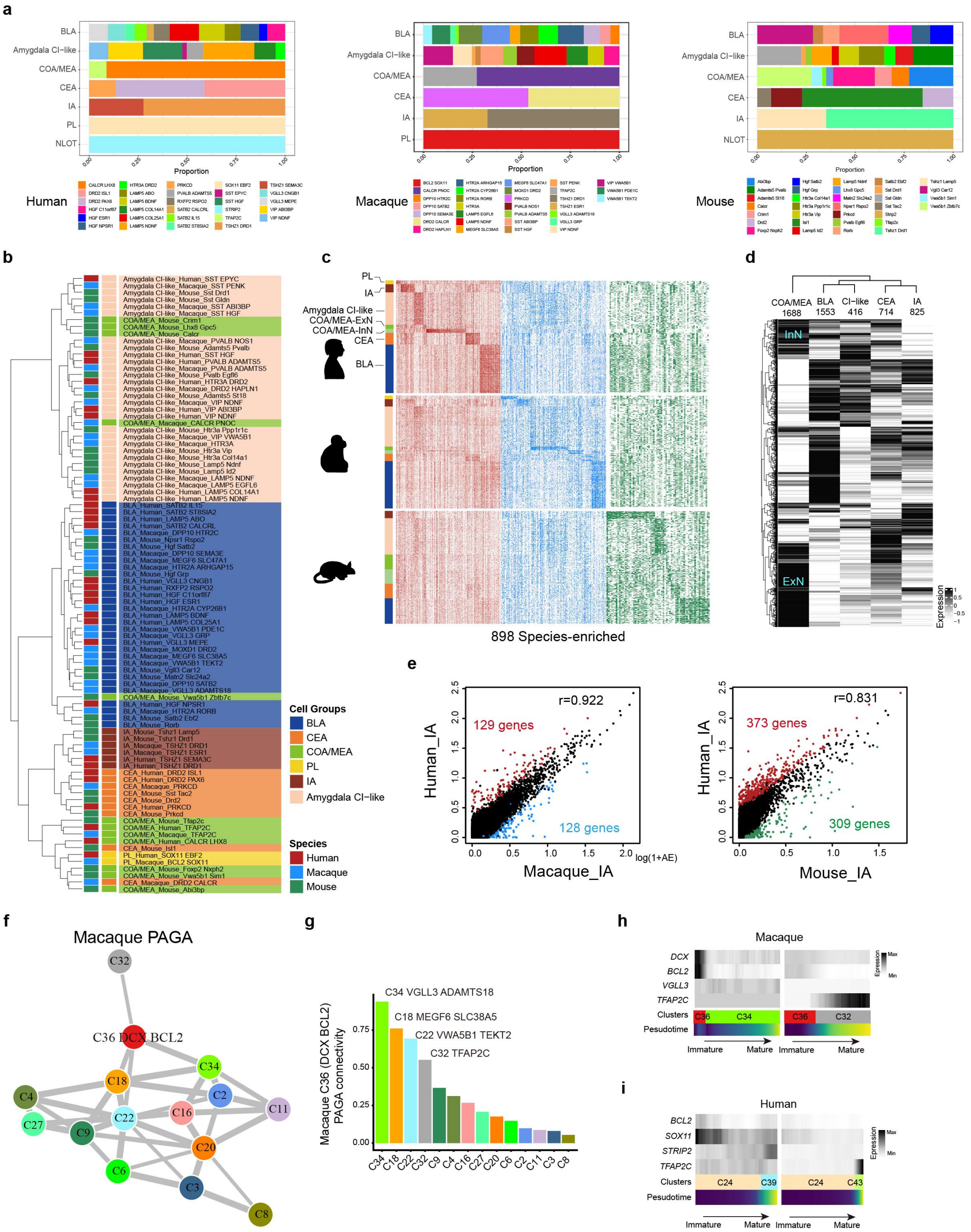

Fig. S4 See next page for caption.

Fig. S4 Characteristics of amygdala subnuclei across species revealed by snRNA-seq, related to Fig. 2

a Bar plots showing proportion of annotated cell types in each cell group in each species.

b Unsupervised hierarchical clustering of cell types from three mammalian datasets based on AUROC scores reported by MetaNeighbor. AUROC scores were used to assess similarities across each pair of cell types.

c Heat map showing expression of species-enriched DEGs, ordered by cell group and species.

d Expression patterns of 5 196 divergent genes (identified between human and mouse, human and macaque). Divergent genes of COA/MEA-ExN and COA/MEA-InN are clustered separately. Top, number of divergent genes restricted to each amygdala cell group.

e Pairwise comparison of log-transformed expression of 13 478 orthologous genes between species in IA subnuclei. Colored genes correspond to significant divergent genes (adjust P-value < 0.01, log2FC > 1). r represents Pearson correlation.

f PAGA graph showing inferred developmental trajectories of macaque excitatory neuronal clusters. Line width represents strength of connectivity between two clusters.

g Bar plot showing PAGA connectivity with Cluster 36 (C36 DCX BCL2) in macaque dataset.

h, i Heat map-reconstructed gene expression changes along PAGA paths for selected signature genes in human (**h**) and macaque (**i**) clusters. Diffusion pseudotime was estimated by Scanpy (v1.8.2).

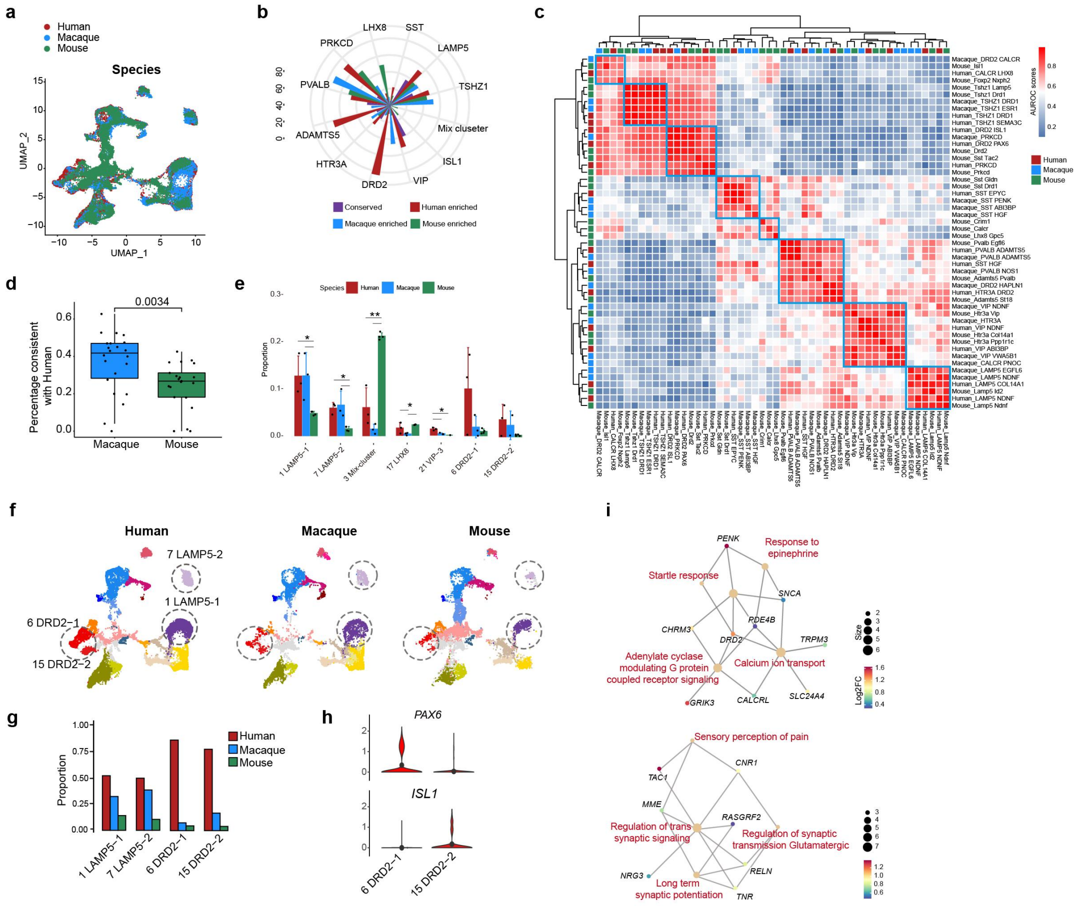

Fig. S5 See next page for caption.

Fig. S5 Cross-species comparisons of inhibitory neurons reveal primate- and human-dominant cell types, related to Fig. 3

a UMAP embedding of integrated inhibitory neuronal datasets (using Seurat), with nuclei colored by species.

b Circular bar plot showing number of conserved and species-enriched DEGs in major cell types from Fig. 3c.

c Heat map showing AUROC scores (estimated by MetaNeighbor) for inhibitory neuronal cell types from three mammals. Cell types with high transcriptomic similarity across three species are marked by blue lines.

d Box plot showing percentage of DEGs in macaque and mouse cell types consistent with humans. Center line, box bounds, and whiskers represent mean, 25th to 75th percentile range, and minimum to maximum range, respectively. Dots represent proportion of consistent DEGs in each pair of cell types. Adjusted *P*-value was calculated by Wilcoxon test, two-side comparison.

e Relative proportions of nuclei in selected cell types in three mammals. ANOVA followed by Tukey's HSD tests were used for multiple comparisons (** adjusted *P*- value < 0.01; * adjusted *P*-value < 0.05). Data are represented as mean ± SEM.

f UMAP integrated inhibitory neuronal datasets, separated by species and cell types, colored as in Fig. 3b. Cell types circled by dotted line are human-dominant clusters.

g Bar plots showing quantification of human-dominant cell types across species.

h Violin plots showing expression of selected genes in DRD2-1 and DRD2-2 clusters. *PAX6* was highly expressed in DRD2-1 cluster and *ISL1* was highly expressed in DRD2-2 cluster.

i Network plots of enriched ontology gene sets and possible functions in DRD2-1 cluster (above) and DRD2-2 cluster (below). Nodes for genes are colored by log2FC and size of pathway nodes represents number of genes.

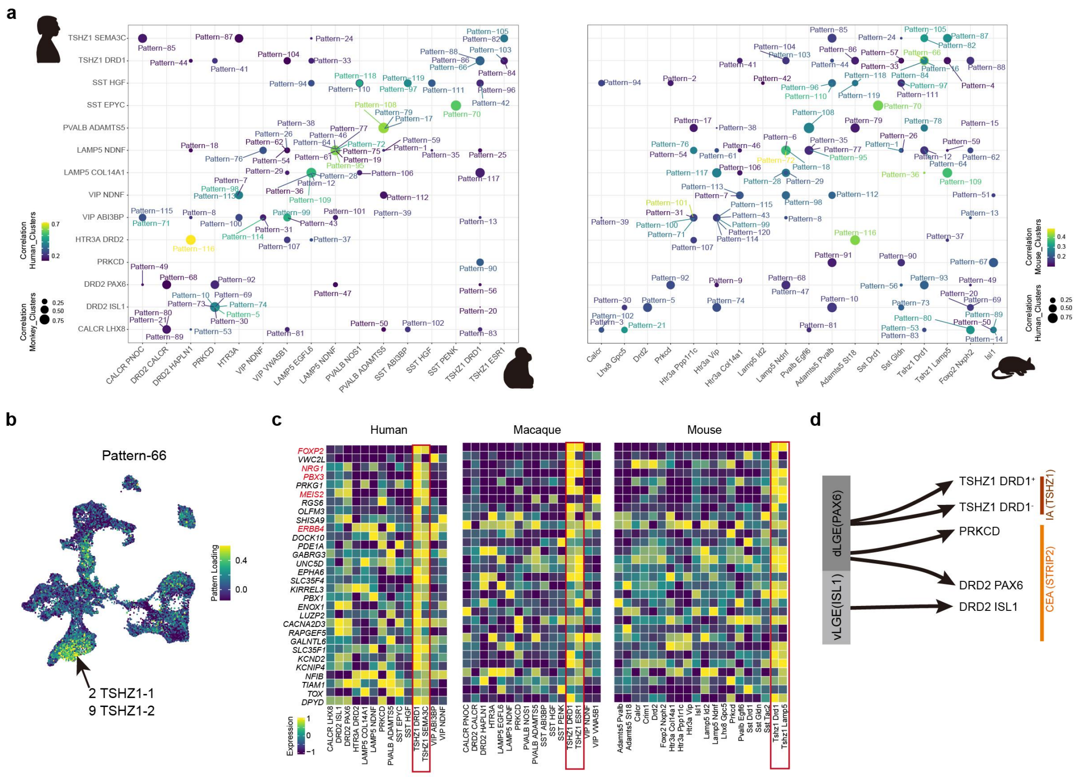

Fig. S6 Cross-species comparisons of inhibitory neurons using CoGAPS reveal conserved and divergent patterns, related to Fig. 3

a Dot plots showing maximally correlated patterns of inhibitory neuronal cell types between humans (y-axis) and macaques (x-axis) and between humans (y-axis) and mice (x-axis). Size of point indicates correlation value for human clusters, and color of point indicates correlation value for macaque or mouse clusters.

b UMAP plot displaying cell loading score of Pattern-66 in (**a**). Pattern-66 was enriched in "TSHZ1" clusters.

c Heat map showing average expression of top 30 weighted genes in Pattern-66 in human, macaque, and mouse inhibitory cell types. Genes are ordered by gene weight. The genes marked in red represent those known to be involved in function and development of *Tshz1* positive IA cell types.

d IA and CEA cell types were assumed to be LGE-derived. IA cell types specifically expressed *TSHZ1* genes, while CEA cell types specifically expressed *STRIP2* genes. dLGE: dorsal LGE; vLGE: ventral LGE.

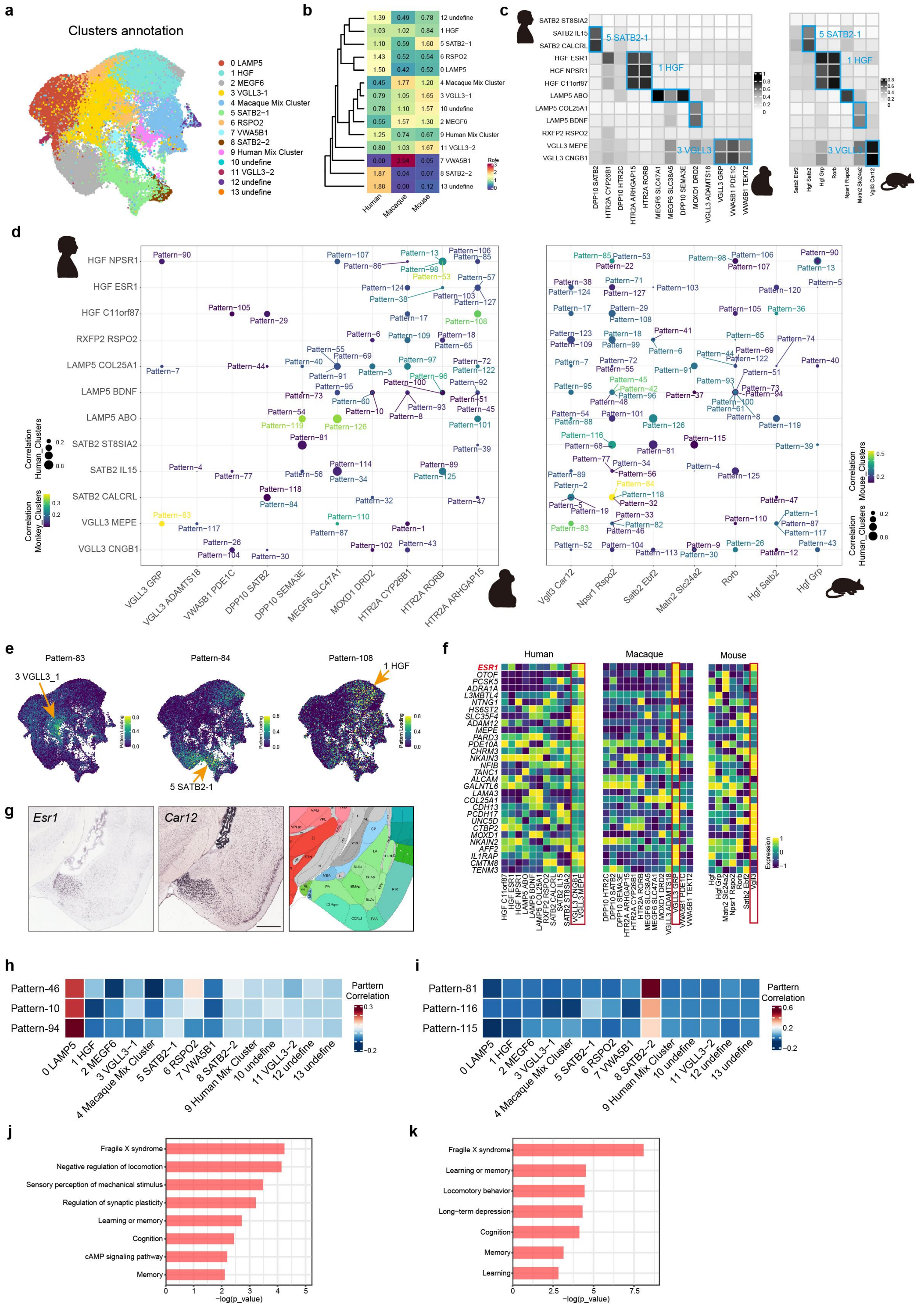

Fig. S7 See next page for caption.

Fig. S7 Cross-species comparisons of BLA excitatory neurons reveal conserved and divergent patterns, related to Fig. 4

a UMAP embedding of integrated BLA excitatory neuronal datasets from three mammals (using Seurat), with nuclei colored by cell type.

b Hierarchical clustering of BLA excitatory neuronal cell types (by *hclust* function). Heat map showing degree of enrichment of each cell type in each species estimated by Ro/e score. The higher the value of Ro/e, the greater enrichment in species.

c Proportion of nuclei overlapping between human and macaque clusters or between human and mouse clusters in integrated datasets. VGLL3, SATB2, and HGF clusters are well conserved.

d Dot plots showing maximally correlated patterns of BLA excitatory neuronal cell types between humans (y-axis) and macaques (x-axis) and between humans (y-axis) and mice (x-axis). Size of point indicates correlation value for human clusters, and color of point indicates correlation value for macaque or mouse clusters.

e UMAP plots displaying cell loading score of Patterns 83, 84, and 108 in (**d**), enriched in "3 VGLL3-1", "5 SATB2_1", and "1 HGF" clusters, respectively.

f Heat map showing average expression of top 30 weighted genes in Pattern-83 in human, macaque, and mouse BLA excitatory neuronal cell types. Genes are ordered by gene weight.

g ISH data from Allen Mouse Brain Atlas showing expression patterns of *Esr1* and *Car12,* scale bar: 300 μm.

h, i Heat map showing correlation of selected Patterns (row) and BLA excitatory neuronal cell types (column).

j, k The significant biological enrichment GO terms for the human-dominant clusters C0 (**j**) and C8 (**k**) pattern genes.

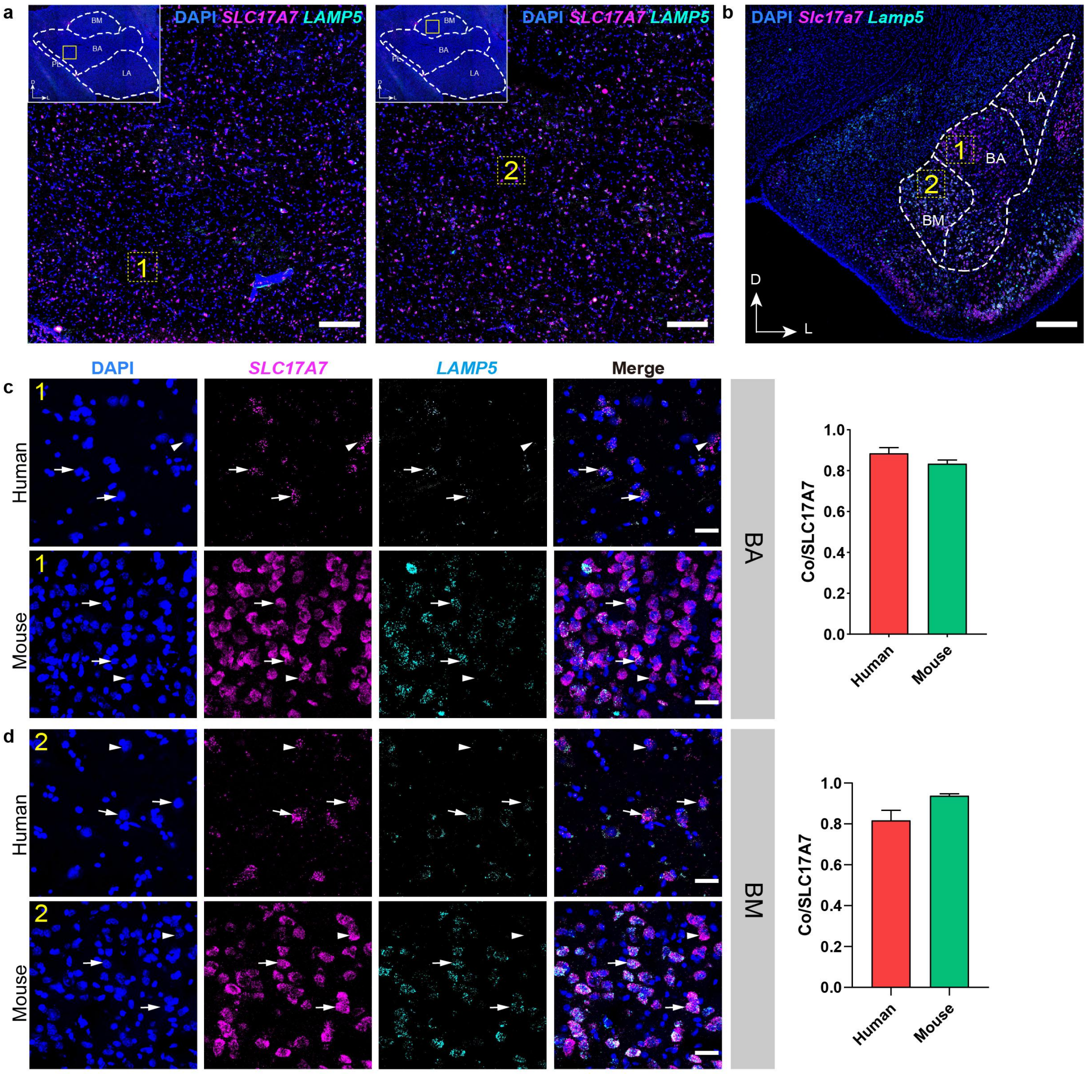

Fig. S8 BA and BM show similar proportions of *LAMP5* positive excitatory neurons, related to Fig. 4

a, b mFISH analysis of *SLC17A7* and *LAMP5* genes in human and mouse BA and BM. Example coronal sections are shown, scale bar: 300 μm. Insets in (**a**), coronal section of entire human amygdala and imaging location. Boxed regions are enlarged in (**c**) and (**d**).

c, d Magnifications of regions in (**a**) and (**b**) and images split into single channels. Arrows indicate *SLC17A7*$^{+}$ *LAMP5*$^{+}$ cells, arrowheads indicate *SLC17A7*$^{+}$ *LAMP5*$^{-}$ cells, scale bar: 30 μm. Proportions of *SLC17A7* and *LAMP5* double-positive cells in total *SLC17A7* positive cells in human and mouse BA and BM are shown on right. Three human and four mouse slices were used for quantification. Unpaired *t*-test was used for significance comparison. Data are represented as mean ± SEM.

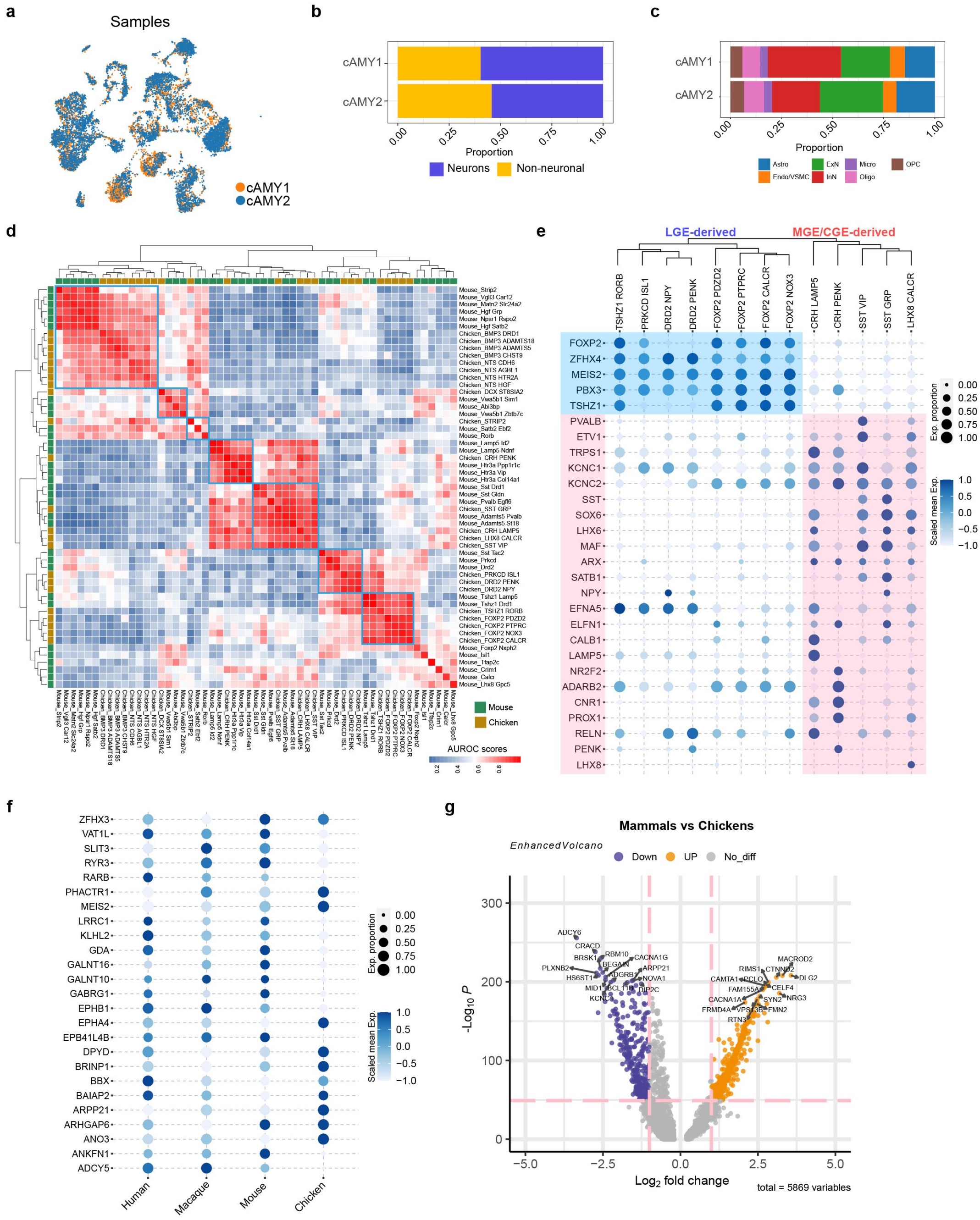

Fig. S9 See next page for caption.

Fig. S9 Evolutionary comparisons of chicken and mouse amygdala cell types and molecular characteristics of assumed CEA neurons between mammals and chickens, related to Fig. 5

a UMAP plot of chicken datasets, with nuclei colored by sample.

b Bar plot showing proportion of neuronal and non-neuronal nuclei in each sample.

c Bar plot showing proportion of major cell classes in each sample.

d Heat map showing AUROC scores (estimated by MetaNeighbor) for mouse and chicken neuronal cell types. Cell types with a high transcriptomic similarity between two species are marked by blue lines.

e Bubble plot showing expression patterns of subpallial embryonic domain marker genes in chicken inhibitory neuronal clusters (marker genes from [23]). Shade of dot represents mean expression within cluster, and size of dot represents percentage of cells within cluster expressing a given gene. LGE, blue; MGE/CGE, red.

f Bubble plot showing that chicken PRKCD and DRD2 clusters also highly expressed most conserved genes of mammal PRKCD and DRD2 clusters. Mammalian conserved genes are from Supplementary Table S4.

g Up and down-regulated genes of mammalian PRKCD and DRD2 clusters compared to corresponding chicken clusters.

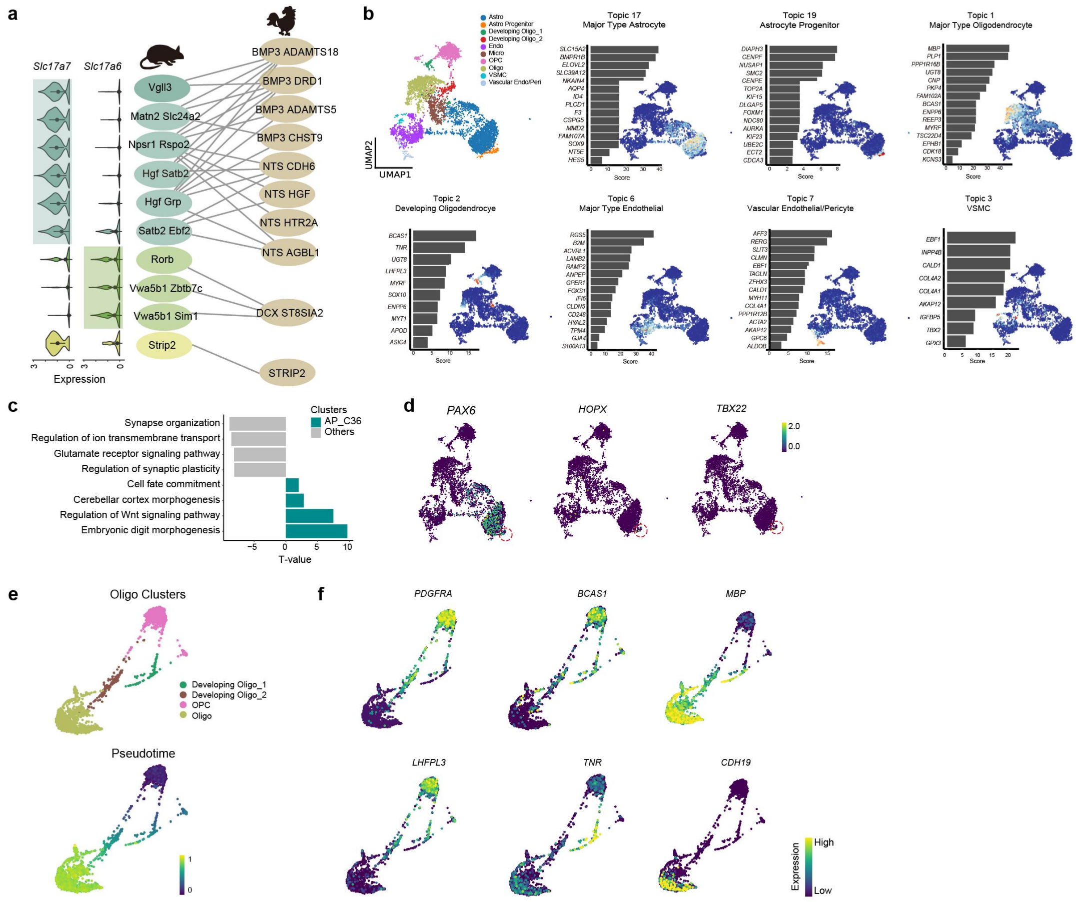

Fig. S10 See next page for caption.

Fig. S10 Characteristics of excitatory and non-neuronal clusters in chicken amygdala, related to Fig. 5

a Correlation analysis of expression profiles between mouse and chicken amygdala excitatory neurons. Lines are displayed between two cell types when the AUROC score is greater than 0.75 (estimated by MetaNeighbor). Expression patterns of *Slc17a7* and *Slc17a6* genes in mouse excitatory neuronal clusters are shown on left.

b Chicken non-neuronal cell-type-associated programs. First panel shows UMAP plot of chicken non-neuronal cell types. For each topic, bar plot shows topic top-weighted gene scores and UMAP plot with nuclei colored by topic loading weight. VSMC: vascular smooth muscle cells.

c Bar plot showing pathways enriched in Cluster 36 astrocyte progenitors (AP_C36) and other clusters, reported by gene set variation analysis (GSVA). *T*-values were computed using limma regression.

d Feature plots showing expression patterns of astrocyte progenitor marker genes in chicken non-neuronal cell types. C36 astrocyte progenitor cluster is marked by red dotted line.

e UMAP plot of chicken OPC and oligodendrocytes, with nuclei colored by cluster (above) and pseudotime (below).

f UMAP plots showing that oligodendrocytes at different maturation levels and OPC expressed different marker genes.

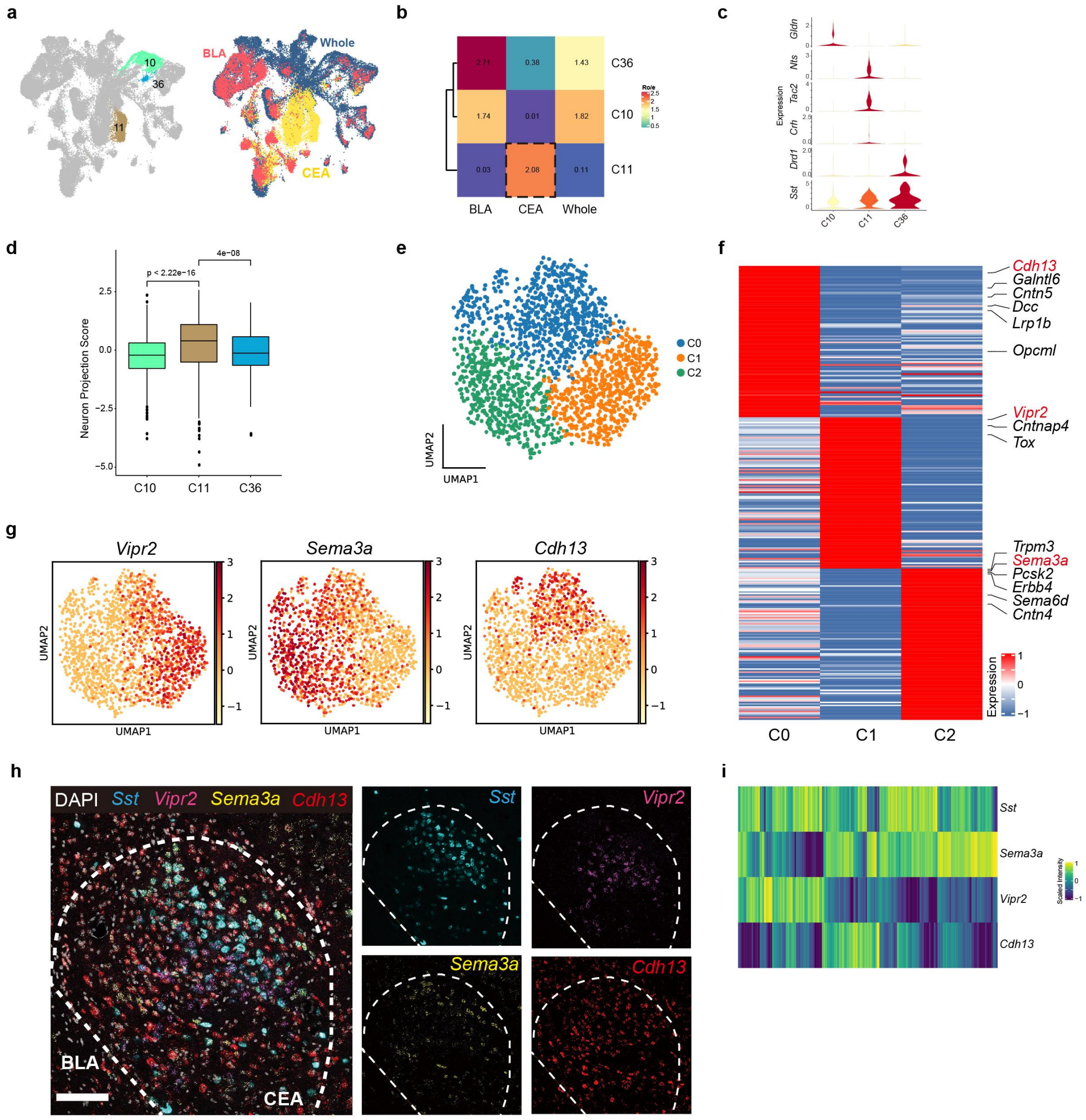

Fig. S11 See next page for caption.

Fig. S11 Subclustering of *Sst*$^+$ cluster in mouse CEA.

a UMAP embedding of mouse datasets. *Sst*$^+$ clusters (left) and dissected areas (right) are highlighted by different colors. Number of clusters is the same as in Supplementary Fig. S2. Whole: whole amygdala.

b Dissection origin of each *Sst*$^+$ cluster estimated by Ro/e score. Cluster marked by dotted lines indicates enrichment in CEA.

c Violin plot showing CEA *Sst*$^+$ cluster with high expression of CEA marker genes *Tac2*, *Nts*, and *Crh*.

d Box plot showing neuron projection signature scores in three *Sst*$^+$ clusters. Adjusted *P*-values were calculated by Wilcoxon test, two-sided comparisons. n = 39 389 nuclei. Center line, box bounds, and whiskers represent mean, 25th to 75th percentile range, and minimum to maximum range, respectively.

e Subclustering of Cluster 11 from (**a**), with nuclei colored by subcluster.

f Heat map showing marker genes for each subcluster, selected genes are labeled on right.

g Feature plots showing marker genes expression in each subcluster.

h mFISH analysis of *Sst*$^+$ subcluster marker genes in mouse CEA, scale bar: 100 µm.

i Heat map showing quantification of mFISH signal intensity in *Sst*$^+$ cells (n = 380). Intensities are scaled by (-1, 1). Cells in heat map columns are ordered by hierarchical clustering.
